# Apigenin accelerates fracture healing by enhancing endochondral differentiation and inflammation resolution via targeting NF-κB signaling

**DOI:** 10.3389/fimmu.2026.1877955

**Published:** 2026-09-14

**Authors:** Haiyu Shen, Guoguo Zhi, Zihao Li, Xueqiao Yan, Zhirui Du, Shaoyang Zhang, Ruichen Yang, Xiuying Ma, Huijuan Liu, Funeng Geng, Baojie Li

**Affiliations:** 1 Institute of Traditional Chinese Medicine and Stem Cell Research, College of Basic Medical Sciences, Chengdu University of Traditional Chinese Medicine, Chengdu, China; 2 Bio-X Institutes, Shanghai Jiao Tong University, Shanghai, China; 3 Periplaneta Americana and Innovative Drugs Key Laboratory of Sichuan Province and Engineering Research Center for Medicinal Animals, Sichuan Good Doctor Panxi Pharmaceutical Co., Ltd, Chengdu, China

**Keywords:** apigenin, fracture healing, inflammation, MSc, NF-κB

## Abstract

**Background:**

Fractures are common yet lack effective pharmacological treatments. Apigenin, a natural flavone, has anti-inflammatory and pro-osteogenic effects. Here, we examined its impact on fracture healing—a multistep process involving hematoma formation, soft callus formation (endochondral differentiation), hard callus formation (osteogenic differentiation), and bone remodeling—and investigated the underlying mechanisms.

**Methods:**

Femoral fracture and calvarial injury models were established to evaluate the effects of apigenin. Local apigenin treatment was initiated at postoperative time points to compare the impact of administration timing. Fracture healing was assessed by radiography, micro-CT, histological analysis, and immunostaining. *In vitro* cultures were performed to examine the effects of apigenin on mesenchymal stem cell (MSC) differentiation. Quantitative PCR, Western blotting, RNA sequencing, and flow cytometry were used to evaluate inflammatory responses and chondrogenic and osteogenic differentiation. *Ikbkb* knockdown and BAY 11–7082 treatment were used to examine the involvement of IKKβ/NF-κB signaling. Molecular docking and molecular dynamics simulations were performed to investigate the potential interaction between apigenin and IKKβ, which was examined by cellular thermal shift assay (CETSA).

**Results:**

Apigenin significantly improved fracture healing when treatment was initiated on postoperative day 5, whereas treatment initiated on day 1 showed no clear therapeutic effect. Apigenin reduced inflammatory responses in the fracture callus and suppressed NF-κB signaling. BAY 11–7082 produced effects similar to those of apigenin in the femoral fracture model, and combined treatment did not produce a clear additional benefit. In BMSCs, apigenin promoted chondrogenic differentiation and attenuated the suppression of chondrogenic markers under inflammatory conditions, whereas its effects on osteogenic differentiation were less consistent. *Ikbkb* knockdown also enhanced chondrogenic differentiation, and addition of apigenin after *Ikbkb* knockdown produced no further clear increase. Molecular docking, molecular dynamics simulations, and CETSA provided supportive evidence for a potential interaction between apigenin and IKKβ.

**Conclusions:**

Through modulation of IKKβ/NF-κB signaling, apigenin acts on both inflammatory responses and MSC differentiation to enhance fracture healing. The therapeutic effect was most evident when treatment was initiated on postoperative day 5, during the transition from inflammation to cartilage formation. These findings suggest that appropriately timed apigenin treatment may represent a potential strategy for improving fracture repair.

## 1 Introduction

The skeletal system provides structural support and enables movement, making maintenance of bone health essential (1). Fractures, commonly caused by trauma, osteoporosis, or other diseases, markedly reduce quality of life and have become a major global health burden due to their contribution to disability and healthcare costs (2, 3). Clinical treatments include surgical fixation and non-surgical approaches such as immobilization and biologics, but these are limited by drug side effects and surgical risks. Pharmacological treatment nonetheless remains essential, particularly in non-operative management and in high-risk patients, to control systemic inflammation and support bone regeneration (4, 5).

Fracture healing is a complex, multistage process that includes hematoma formation, cartilaginous callus formation, hard callus formation, and remodeling (6–9). After injury, neutrophils and macrophages infiltrate the hematoma within 2 days (1). This inflammatory phase is essential for successful bone repair because it clears debris, and inflammatory cytokines such as TNF-α and IL-1β promote mesenchymal stem cell recruitment and initiate cartilaginous callus formation (1). This is followed by a phase of inflammation resolution, which is required for successful fracture healing. However, excessive or prolonged inflammation can lead to delayed healing or non-union (10). In fracture healing, MSCs regenerate bone either by directly differentiating into osteoblasts (intramembranous ossification) or by first becoming chondrocytes (endochondral ossification).

The NF-κB and JAK-STAT pathways are critical regulators of inflammation, which drive cytokine expression in immune cells. The NF-κB pathway also plays a complex role in skeletal development and bone regeneration. Under physiological conditions, canonical NF-κB (via p65/RelA) supports bone growth by promoting osteoblast proliferation and differentiation and limiting apoptosis (11). In pathological states, however, NF-κB activation suppresses chondrogenesis and osteogenesis, whereas its inhibition upregulates osteogenic matrix genes (Runx2, Sp7, osteocalcin) and enhances bone formation (12–14). RelB-dependent non-canonical NF-κB signaling also negatively regulates bone formation at fracture sites (15). Thus, time-dependent modulation of NF-κB within the fracture microenvironment represents a key therapeutic strategy to improve healing.

Traditional Chinese Medicine has a long history of use in bone disorders, especially osteoporosis (16–21). Apigenin (API), a natural flavone, has been investigated as a potential therapeutic agent for osteoporosis and osteoarthritis, owing to its potent anti-inflammatory and antioxidant properties (19, 20, 22–24). API may suppress inflammation by targeting the JAK–STAT pathway, NF-κB signaling, inflammasomes, and other related molecules (25–28). API mitigates bone loss by stimulating diverse signaling cascades, ranging from Wnt/β-catenin and MAPK pathways to SIRT1/HIF-1 axes (29–34). There are also studies indicating that API may exert inhibitory or even cytotoxic effects on osteogenesis under certain conditions (35). Apigenin, administered in later stages, has been reported to promote bone fracture healing in rats by activating Wnt/β-catenin signaling and upregulating Runx2 and Smad1, thereby enhancing MSC osteogenic differentiation (36–39). However, it remains unclear how apigenin integrates its immunosuppressive effects during both the initiation and resolution of inflammation with its regulation of MSC fate toward endochondral versus osteogenic differentiation, both of which are critical for effective fracture healing (1, 38).

This study aimed to investigate the therapeutic effects of apigenin on early stages of fracture healing and to elucidate the mechanisms by which apigenin exerts these effects.

## 2 Materials and methods

### 2.1 Animals

All animal procedures were conducted in compliance with the Guide for the Care and Use of Laboratory Animals (National Research Council) and approved by the Institutional Animal Care and Use Committee of Shanghai Jiao Tong University (approval number A2024014-001) on October 12, 2024. Male C57BL/6 mice (12 weeks of age) were sourced from the Southern Model Organisms Research Center and maintained within the Specific Pathogen Free (SPF) vivarium at Shanghai Jiao Tong University. For therapeutic intervention, API (45 ng/kg or 5 mg/kg) or an identical volume of the vehicle mixture (DMSO/PEG300/Tween 80/ddH_2_O) was delivered via local injection on alternate days until the study endpoint.

### 2.2 Model

Femur fracture model.

Closed mid-diaphyseal femoral fractures were induced in mice under intraperitoneal sodium pentobarbital anesthesia (40 mg/kg). A medial knee incision was made, and a 27-gauge needle was inserted into the intramedullary canal for fixation. Fractures were then generated using a three-point bending device (a 500-g blunt blade dropped from 20 cm) and confirmed radiographically.

Mice were randomized into treatment cohorts (n = 5 per group). In the day-1 treatment cohort, mice received either vehicle or high-dose API (5 mg/kg). In the day-5 treatment cohort, mice received vehicle, low-dose API (45 ng/kg), or high-dose API (5 mg/kg). An additional cohort received vehicle or API (5 mg/kg) beginning day 2 after fracture; callus tissue was collected on day 7 for flow-cytometric and RT-qPCR analyses.

For NF-κB inhibition experiments, BAY 11-7082 (5 mg/kg) was administered locally at the fracture site beginning on day 5, either alone or in combination with API. All treatments were administered locally on alternate days until the respective experimental endpoints.

Calvarial injury model.

Critical-sized calvarial defects (2 mm in diameter) were surgically created in mice under intraperitoneal sodium pentobarbital anesthesia (40 mg/kg). After shaving and disinfecting the surgical site, a midline longitudinal incision was made over the scalp to expose the sagittal suture. The periosteum was gently reflected to uncover the parietal bones. Using a low-speed dental drill with a trephine bur, two bilateral full-thickness defects were created, one in each parietal bone, while preserving the integrity of the underlying dura mater. After removal of the bone discs and irrigation to clear debris, the wound was closed in layers with sutures. For the calvarial defect experiment, mice were randomly assigned to vehicle or API (5 mg/kg) treatment groups, with treatment initiated on postoperative day 5. The exact sample size for each analysis is indicated in the corresponding figure legend.

### 2.3 X-Ray and micro-CT analysis

Bone radiographs (normal and fractured) were acquired using a Cabinet X-ray system (LX-60, Faxitron Bioptics) with standardized settings (45 kV, 8 s) on days 7, 14, and 21 post-fracture. To quantitatively monitor temporal changes in callus formation and subsequent remodeling, we calculated the callus index from serial radiographs. The callus index was defined as the ratio of the maximum external diameter of the fracture callus to the external diameter of the adjacent intact diaphysis. Femoral specimens were scanned using a SkyScan 1176 micro-CT scanner (Bruker) according to the manufacturer’s instructions. Scans were performed at 8.96 μm voxel size, 45 kV, 500 μA, with 0.6° rotation steps over 180°. Three-dimensional reconstructions were generated, and BV/TV (%) and Tb.N (mm^-^¹) were quantified for microarchitectural analysis. The region of interest was defined around the fracture callus.

### 2.4 Tissue preparation and histology

Samples were collected at 7, 14, and 21 days after fracture. Tissues were fixed in 4% paraformaldehyde (Aladdin, Shanghai, China) for 24 h at room temperature, then decalcified in decalcifying solution at room temperature, with the solution changed every 7 days for a total of four changes.

### 2.5 Safranin O staining

The decalcified bone tissue was embedded in paraffin, and femoral specimens were sagittally sectioned at 6 μm thickness and stained with Safranin O for histological analysis. Sections were examined using an Olympus microscope (Olympus, Tokyo, Japan), and fracture healing was assessed with a standardized histological scoring system.

### 2.6 Immunofluorescence and immunohistochemical staining

For immunostaining, sections were permeabilized with 0.1% Triton X-100 for 30 min, then subjected to antigen retrieval in citrate buffer at 89 °C for 20 min and cooled. Sections were blocked with 10% goat serum for 40 min, incubated with primary antibodies overnight at 4 °C, and then with secondary antibodies for 30 min at 37 °C. Antibodies included p-p65 (CST, 3033), CD45 (Abcam, ab40763), Ly6G (BioLegend, 127601), RUNX2 (CST, 12556), PCNA (Santa Cruz, sc-56), Sox9 (Millipore, AB5535), OCN (Abcam, ab93876), B220 (Invitrogen, 2340525), CD3 (abcam, ab16669). Slides were mounted with antifade medium containing DAPI (Thermo Fisher Scientific), and images were acquired using an Olympus DP72 microscope.

For immunohistochemical staining, sections were subjected to antigen retrieval and blocking, followed by incubation with the indicated primary antibodies and appropriate secondary antibodies. Signals were visualized using DAB and counterstained with hematoxylin. Images were acquired using an Olympus DP72 microscope.

### 2.7 Flow cytometry analysis

Callus tissue was harvested from mice and digested with type II collagenase. The resulting cell suspension was passed through a 70-μm cell strainer to generate a single-cell suspension. Dead cells were identified using a LIVE/DEAD Fixable Dead Cell Stain Kit and excluded from subsequent analysis. Cells were stained with fluorophore-conjugated antibodies on ice for 1 h. The following antibodies were used at the indicated dilutions: CD11b-BV421 (BioLegend, Cat. No. 108105, clone D7; 1:50), CD45-PE594 (BioLegend, Cat. No. 103145, clone 30-F11; 1:200), F4/80-FITC (BioLegend, Cat. No. 123107, clone BM8; 1:200), Ly6G-BV711 (BioLegend, Cat. No. 127209, clone TY/11.8; 1:200), and CD206 (BioLegend, Cat. No. 141708, clone C068C2; 1:200). Single-stained controls were used for fluorescence compensation. Samples were acquired on a BD FACSAria II flow cytometer using FACSDiva software (v6.1.3), and data were analyzed using FlowJo (v10.4). Debris and doublets were excluded based on forward- and side-scatter characteristics and FSC-A/FSC-H gating, respectively.

### 2.8 Local pharmacokinetic analysis of apigenin in callus

Local pharmacokinetic analysis was performed after local apigenin administration. Fracture callus samples were collected at 2, 4, 8, and 24 h after dosing. Approximately 50 mg of callus tissue was extracted with 300 μL of methanol/acetonitrile/water (2:2:1, v/v/v) and homogenized at 60 Hz for 2 min. The extract was collected and concentrated to dryness by centrifugal evaporation. The dried residue was reconstituted in 100% methanol and subjected to LC–MS/MS analysis using an ACQUITY UPLC H-Class system coupled to a Xevo TQ-XS mass spectrometer. Data were processed using MassLynx software. Apigenin concentrations were determined from integrated chromatographic peak areas using a calibration curve generated with reference standards and were expressed as ng/g of tissue.

### 2.9 *In vitro* cell differentiation assays

Long bones from the limbs of wild-type C57BL/6 mice (10–12 weeks old) were dissected free of muscle and cartilage and rinsed in PBS. Bone marrow was flushed out with α-MEM using a 1-ml syringe, centrifuged, and plated at 2 × 10^7^ cells/mL in α-MEM with 10% FBS. Cells were incubated for 24 h at 37 °C, 5% CO_2_, then the medium was replaced with α-MEM containing 2 mM L-glutamine, 100 U/mL penicillin and 100 μg/mL streptomycin, and 10% FBS to remove non-adherent cells; medium was changed every 3–4 days. After 2 weeks, the adherent bone marrow-derived stromal cells showed positive immunofluorescence staining for vimentin.

### 2.10 Cell viability was assessed using the CCK-8 assay

BMSCs were seeded in 96-well plates at 5 × 10³ cells per well in 100 μL α-MEM containing 10% FBS and incubated for 24 h. The medium was then replaced with α-MEM containing API or vehicle. CCK-8 measurements were performed in parallel wells at 0, 24, and 48 h after treatment. At each time point, 10 μL of CCK-8 solution was added to each well, followed by incubation at 37 °C for 2 h. Absorbance at 450 nm was measured using a Tecan Spark 10M microplate reader (Tecan, Shanghai, China). Wells containing α-MEM and CCK-8 solution without cells were used as blanks. Relative cell viability was calculated by normalizing the absorbance of API-treated cells to vehicle-treated cells at the corresponding time point.

### 2.11 Chondrogenic differentiation induction and alcian blue staining

For chondrogenic differentiation, BMSCs were resuspended at 1.6 × 10^5^ cells/mL, seeded in 12-well plates, and incubated at 37 °C. The next day, cells were cultured in α-MEM with 10% FBS, 100 nM dexamethasone, 10 ng/mL TGF-β1, 1 mM vitamin C phosphate, and antibiotics, with medium changes every 3 days for 21 days, then fixed in 4% paraformaldehyde and stained with Alcian blue. Alcian blue specifically labels cartilage matrix by binding acidic mucopolysaccharides. For API treatment, cells were cultured in chondrogenic medium with 0.1 or 1 μM API for 21 days, fixed, stained with Alcian blue for 30 min, washed with PBS, and imaged using an inverted microscope with a digital camera.

For experiments under inflammatory conditions, BMSCs were treated with 100 ng/mL LPS from the beginning of chondrogenic induction, with or without 1 μM API. LPS and API were maintained throughout the 14-day induction period and replenished at each medium change. Total RNA was collected on day 14 for RT-qPCR analysis.

### 2.12 Osteogenic differentiation and alkaline phosphatase staining

For osteoblast differentiation, BMSCs were seeded in 12-well plates at 5 × 10^4^ cells per well in complete medium. The next day, they were cultured for 7 days in osteogenic medium containing 10 mM β-glycerophosphate, 100 nM dexamethasone, and 50 μg/mL ascorbic acid, with medium changes every 3 days. After 7 days in osteogenic medium with API (0.1 or 1 μM), cells were washed twice with PBS, fixed in 4% PFA for 20 min, and stained with an ALP kit (1 mL/well; Jinqiao, Shanghai, China) for 10 min at 37 °C. ALP-positive nodules were imaged using an inverted microscope with a digital camera.

For experiments under inflammatory conditions, BMSCs were treated with 100 ng/mL LPS from the beginning of osteogenic induction, with or without 1 μM API. LPS and API were maintained throughout the 7-day induction period and replenished at each medium change. Total RNA was collected on day 7 for RT-qPCR analysis.

### 2.13 Bulk RNA-seq

Bulk RNA-seq was performed on fracture callus tissue. Total RNA was extracted from fracture callus tissue using TRIzol reagent (Invitrogen, Carlsbad, CA, USA). Briefly, callus tissue was ground under liquid nitrogen and homogenized in TRIzol. Following phase separation with chloroform/isoamyl alcohol, the aqueous phase was collected, and RNA was precipitated with isopropanol, washed with 75% ethanol, and dissolved in RNase-free water. RNA quantity and integrity were assessed using a Fragment Analyzer and an Agilent 2100 Bioanalyzer (Agilent Technologies, Santa Clara, CA, USA). Total RNA was treated with DNase I to remove residual genomic DNA, and ribosomal RNA was depleted using an RNase H-based method. The rRNA-depleted RNA was fragmented, followed by first- and second-strand cDNA synthesis. The resulting cDNA was purified using magnetic beads, end-repaired, A-tailed, and ligated to indexed adapters. After PCR amplification and purification with AMPure XP beads, library quality was assessed using an Agilent 2100 Bioanalyzer. Double-stranded PCR products were subsequently heat-denatured and circularized to generate single-stranded circular DNA, which was amplified by phi29 DNA polymerase to generate DNA nanoballs (DNBs). DNBs were loaded onto a patterned nanoarray, and single-end 50-bp reads were generated on the BGISEQ-500 platform.

### 2.14 Processing of bulk RNA-seq data

SOAPnuke (v1.4.0) was used to filter raw sequencing data and generate FASTQ files, with low-quality reads (defined as those with >20% of bases having a quality score <15) and reads with more than 5% unknown bases (N) removed. Clean reads were aligned to the mouse reference genome GRCm38.p6 using Bowtie2 (v2.2.5), and gene-level counts were obtained with RSEM (v1.3.1). A total of 6 samples (3 biological replicates per condition) were sequenced, with an average yield of 6.72 Gb of clean data per sample and alignment rates of 99.11% (genome) and 76.18% (gene set). Differential expression analysis was performed with DESeq2 (v1.30.1) in R, using thresholds of |log_2_FC| ≥ 1 and FDR ≤ 0.05. Principal component analysis was performed with the prcomp function, and heatmaps were generated using the pheatmap package (v1.0.12) from scaled sample-by-gene matrices.

### 2.15 GO and KEGG analysis

Differentially expressed genes were analyzed for Gene Ontology (GO) term and Kyoto Encyclopedia of Genes and Genomes (KEGG) pathway enrichment using the clusterProfiler R package (v3.18.1). A q value of ≤ 0.05 was considered statistically significant.

### 2.16 Molecular docking and molecular dynamics simulations

Two-dimensional structures of small-molecule ligands were downloaded from PubChem and converted to three-dimensional (3D) structures in PyMOL. The 3D structures of receptor proteins were obtained from the RCSB Protein Data Bank and processed in PyMOL to remove water molecules and bound ligands. Receptors and ligands were then prepared, and grid boxes were defined using AutoDock Tools (v1.5.6). Molecular docking was performed with AutoDock Vina (v1.1.2), and docking poses were visualized in PyMOL.

Molecular dynamics (MD) simulations were performed using GROMACS 2022. The protein was parameterized using the AMBER14SB force field, while the ligand topology was generated using Sobtop 1.0 (dev3.1) with the GAFF2 force field. Ligand atomic charges were assigned using the restrained electrostatic potential (RESP) method. The protein–ligand complex was solvated in a cubic box of TIP3P water, and Na^+^ and Cl^-^ ions were added to neutralize the system and establish a final NaCl concentration of 0.15 M. Long-range electrostatic interactions were calculated using the particle mesh Ewald (PME) method. Short-range interactions used a 1.0-nm cutoff, and bond lengths were constrained using the LINCS algorithm.

Before production simulations, the system underwent energy minimization with 3,000 steps of steepest-descent minimization followed by 2,000 steps of conjugate-gradient minimization. The system was subsequently equilibrated and simulated under NPT conditions at 310 K and 1 bar using the Nosé–Hoover thermostat and Parrinello–Rahman barostat, respectively. Simulations were performed with a 2-fs integration time step for 100 ns. Trajectories were analyzed using GROMACS tools to calculate root-mean-square deviation (RMSD), root-mean-square fluctuation (RMSF), hydrogen-bond occupancy, and radius of gyration (Rg).

### 2.17 Reverse transcription quantitative polymerase chain reaction

After treatment, total RNA was extracted from the indicated cell or tissue samples using TRIzol reagent containing RNase inhibitors. RNA was reverse-transcribed with the PrimeScript RT kit, diluted 1:10, and analyzed by qPCR on a Roche LightCycler 480 II, with target gene expression normalized to GAPDH or B2M for comparison between groups (**Supplementary Table 1**).

### 2.18 Western blotting analysis

The callus tissue was harvested from the fracture site. Samples were homogenized in T-PER tissue protein extraction buffer (Thermo, 78510) containing 1 mM PMSF and protease inhibitors (1 μg/mL aprotinin, leupeptin, and pepstatin); cell lysates were prepared in TNEN buffer with the same inhibitors. Protein concentration was measured by Bradford assay (Bio-Rad), followed by SDS-PAGE and transfer to PVDF membranes (Millipore). Immunoblotting was performed with antibodies against p-p65 (3033S, 1:1000), total p65 (8242, 1:1000), total IKKβ (8943, 1:1000) from Cell Signaling Technology, and GAPDH (sc-81178, 1:1000), β-actin (sc-47778, 1:1000) from Santa Cruz. Band intensities were quantified by densitometry using ImageJ.

### 2.19 SiRNA transfection procedure

BMSCs were transfected with an siRNA targeting mouse *Ikbkb* (5′-GGGAUCACCUCAGAUAAAUTT-3′) or a nonspecific negative-control siRNA (si-NC; GenePharma, Shanghai, China). Transfections were performed using siRNA-Mate SUS reagent at a final siRNA concentration of 50 nM.

After 24 h, the transfection medium was replaced with chondrogenic or osteogenic induction medium containing 1 μM API or vehicle. Knockdown efficiency was confirmed by RT-qPCR and Western blotting before differentiation experiments.

### 2.20 Cellular thermal shift assay

RAW264.7 cells were harvested, resuspended as single-cell suspensions, and lysed by repeated freeze–thaw cycles to prepare total protein lysates. Lysates were incubated with 50 μM apigenin or an equal volume of DMSO (vehicle control) for 1 h at room temperature.

Samples were then heated at temperatures ranging from 37 to 57 °C for 5 min and allowed to cool to room temperature. Following centrifugation, supernatants were collected and analyzed by Western blotting to assess IKKβ protein levels.

### 2.21 Data and statistical analyses

All experiments were designed and analyzed in accordance with published pharmacological guidelines. Data are presented as mean ± SD or SEM, as indicated, and were normalized when appropriate. Mice were randomly assigned to experimental groups; however, investigators were not formally blinded to treatment allocation. No animals or samples were excluded from the analyses unless otherwise stated. For histological analyses, five microscopic fields per mouse were quantified and averaged. No formal *a priori* power calculation was performed. Group sizes were selected on the basis of previous studies using comparable fracture models and preliminary experiments, and the exact sample size for each group is reported. Immunofluorescence, RT-qPCR, and Western blot data were analyzed using GraphPad Prism 10.0. Comparisons between two groups were performed using unpaired, two-tailed Student’s *t*-tests. Experiments involving more than two groups were analyzed using one- or two-way ANOVA, as appropriate. A *P* value < 0.05 was considered statistically significant.

## 3 Results

### 3.1 The therapeutic effect of API on femoral fracture healing

The first used a murine femoral fracture model to evaluate the effects of API on fracture healing. Healing in this model proceeds through four overlapping phases: hematoma formation, cartilaginous callus formation, bony callus formation, and bone remodeling. Previous studies have evaluated two locally administered doses of API: 45 ng/kg (37) and 5 mg/kg (40), each in a 20-μL volume. Local administration of API at 45 ng/kg, initiated 7 days after fracture and continued for 4–6 weeks, promoted fracture healing (37). This treatment period primarily encompasses the late cartilaginous-callus and bony-callus formation phases (1). We therefore tested whether this same dose would improve fracture healing in our settings. However, local administration of API at 45 ng/kg beginning on day 5 did not improve healing when assessed 21 days after fracture (**Figure 1** and **Supplementary Figure 1**), likely because the dose was too low.

**Figure 1:**
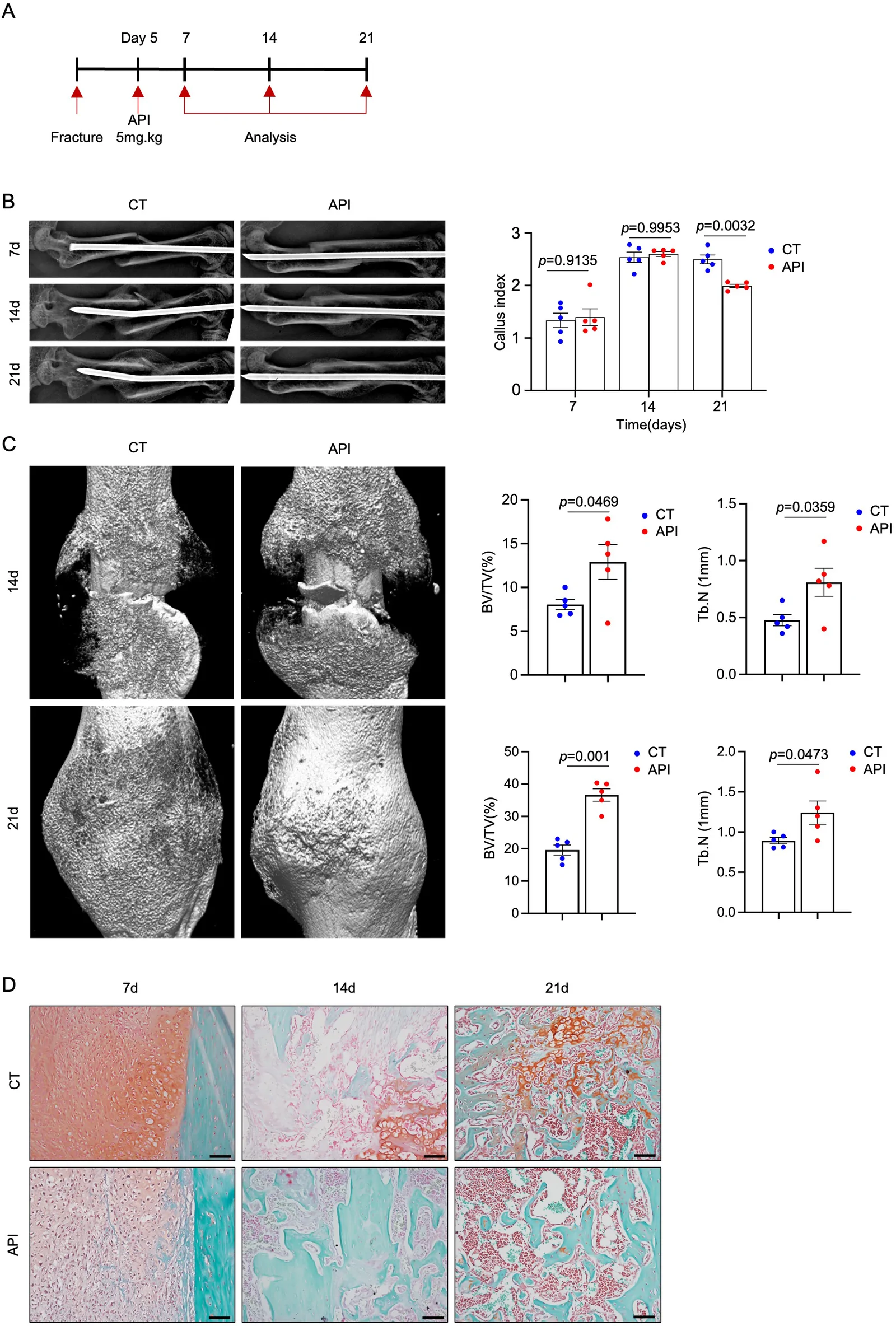
Apigenin promotes fracture healing in a mouse femoral fracture model. **(A)** Schematic of the femoral fracture procedure and local API administration. **(B)** Representative longitudinal X-ray images showing callus formation in control (CT) and API groups at 7, 14, and 21 days, with Callus Index quantified in the bar graph (n = 5). **(C)** Micro-CT 3D reconstructions of the fracture site at 14 and 21 days, with bar graphs comparing BV/TV and Tb.N between CT and API groups at 14 days (BV/TV: P = 0.0469; Tb.N: P = 0.0359) and 21 days (BV/TV: P = 0.0002; Tb.N: P = 0.0473) (n = 5). **(D)** Safranin O/Fast Green staining of fracture callus at 7, 14, and 21 days, showing enhanced endochondral ossification and increased new bone formation in the API group relative to control (n = 5). Data are presented as mean ± SEM. Callus Index data in B were analyzed using one-way ANOVA, whereas comparisons in C were performed using an unpaired two-tailed Student’s *t*-test. Scale bars: 50 μm.

We next evaluated API at 5 mg/kg. When treatment was initiated on postoperative day 1 and fracture healing was assessed on day 21, API had no significant effect (**Supplementary Figure 1**). Similarly, when API treatment was initiated on day 2 and callus tissue was collected on day 7, expression of the chondrogenic markers *Sox9* and *Col10a1* and the osteogenic markers *Runx2* and *Alpl* did not differ significantly between API-treated and control groups (**Supplementary Figure 1**). In contrast, initiating API treatment at 5 mg/kg on postoperative day 5 markedly improved fracture healing. Radiographs obtained on day 21 showed a less distinct fracture line and reduced callus size. Consistent with more advanced callus maturation and remodeling, the callus index was significantly lower in API-treated mice than in controls (**Figure 1**). Micro-CT analysis revealed that BV/TV and Tb.N were significantly increased at the fracture site compared with controls (**Figure 1**). Histological analysis using Safranin O/Fast Green and H&E staining further showed enhanced endochondral ossification and increased new bone formation in the API-treated group (**Figure 1**). Together, these findings indicate that the effects of API on fracture repair depend strongly on treatment timing.

### 3.2 Local pharmacokinetics of API in the fracture callus

To assess the local pharmacokinetics of API, we administered a single local injection of API (5 mg/kg) at the fracture site. Fracture-callus samples were collected at 2, 4, 8, and 24 h after injection, and API concentrations were quantified by LC–MS/MS (**Supplementary Figure 1**). API concentrations started at approximately 7,500 ng/g at 2 h and then gradually declined; however, API remained detectable in callus tissue at 24 h.

### 3.3 API showed minimal effect on the healing of calvarial bone injury

A previous study showed that API could promote healing of bone injuries on the calvaria in rats (36). This injury model represents intramembranous ossification of MSCs, without endochondral ossification. We repeated the experiment in this mouse model using 5 mg/kg apigenin. After 28 days, micro-CT 3D analysis showed that the defect area remained largely unchanged with apigenin treatment, with only minor closure (**Supplementary Figure 2A**). Quantitative analysis indicated that apigenin had no significant effect on calvarial bone regeneration compared with vehicle-treated controls (**Supplementary Figure 2**). Immunostaining showed no significant differences in the protein levels of the osteogenic markers RUNX2 and OCN between API-treated and control mice (**Supplementary Figure 2**). These findings suggest that, at this dose, and under these experimental conditions, API did not significantly improve calvarial defect repair. Because this model heals primarily through intramembranous ossification without a chondrogenic phase, these findings suggest that API exerts a greater therapeutic effect in the endochondral-dominant femoral fracture model.

### 3.4 Transcriptomic profiling reveals API’s effects on inflammatory and chondrogenic pathways

To elucidate the molecular mechanisms by which API influences fracture healing, RNA sequencing (RNA-seq) was performed on fracture callus tissues (n=3) collected at day 7 post-fracture. On average, we obtained 6.72 Gb of clean data per sample. Of these reads, 99.11% aligned to the reference genome, and 76.18% mapped to annotated genes, enabling detection of 18,005 genes overall. Compared with controls, the API-treated group showed 1,589 upregulated and 220 downregulated genes (**Figure 2**). Pathway enrichment analysis showed that genes downregulated by API were enriched in inflammation-related pathways, including NF-κB signaling, chemokine signaling, Fcγ receptor–mediated phagocytosis, and HIF-1 signaling (**Figure 2** and **Supplementary Figure 3**), whereas genes upregulated by API were enriched in processes related to endochondral ossification, positive regulation of chondrocyte differentiation, and cartilage development (**Figure 2**).

**Figure 2:**
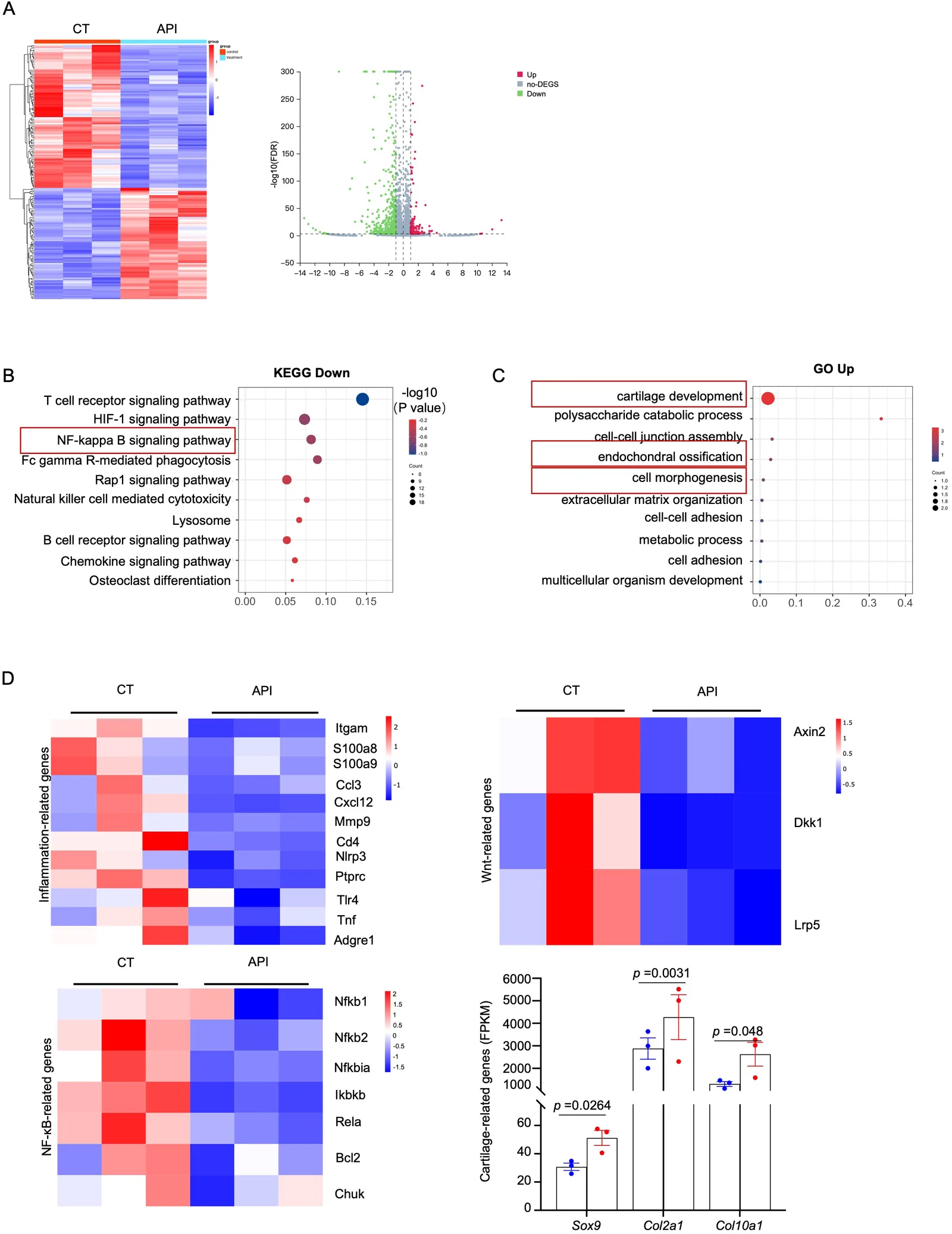
Characteristics of the transcriptional profile of early fracture callus. **(A)**. Heatmap of differentially expressed genes in fracture callus from control and API-treated mice (n = 3 biological replicates per group). **(B, C)**. KEGG and GO enrichment analyses of upregulated and downregulated genes in API-treated callus versus control. **(D)**. Expression of genes related to inflammation, Wnt/β-catenin and NF-κB signaling, and cartilage (FPKM). Differentially expressed genes were defined as |log_2_FC| ≥ 1 and FDR ≤ 0.05.

API treatment broadly reduced the expression of inflammatory mediators, including *Ptprc*, *Itgam*, *Tnf*, and the inflammasome component *Nlrp3*, while increasing the expression of chondrogenic genes such as *Sox9*, *Col2a1*, and *Col10a1* (**Figure 2**). We next examined signaling pathways potentially involved in these changes. The transcriptional profile of the canonical Wnt/β-catenin pathway did not show clear evidence of activation at day 7. For example, *Lrp5* and *Axin2* were downregulated, despite reduced expression of the Wnt antagonist *Dkk1* (**Figure 2**). In contrast, API markedly downregulated several components of the canonical NF-κB pathway, including *Rela*, *Nfkb1*, *Ikbkb* (encoding IKKβ), and *Chuk* (encoding IKKα) (**Figure 2**). Expression of JAK–STAT pathway genes was also largely unchanged (**Supplementary Figure 3**). These findings led us to further examine IKK/NF-κB signaling as a potential mediator of the effects of API.

### 3.5 API mitigates the *in vivo* inflammatory response at the fracture site

We further characterized the inflammatory response in fracture callus tissue by immunofluorescence, flow cytometry, and RT-qPCR. Compared with controls, API-treated mice showed markedly fewer CD45^+^ leukocytes and Ly6G^+^ neutrophils (**Figure 3A, B**). RT-qPCR showed reduced expression of *Il1b* and *Tnf* and increased expression of *Il10* in the API-treated group (**Figure 3C**).

**Figure 3:**
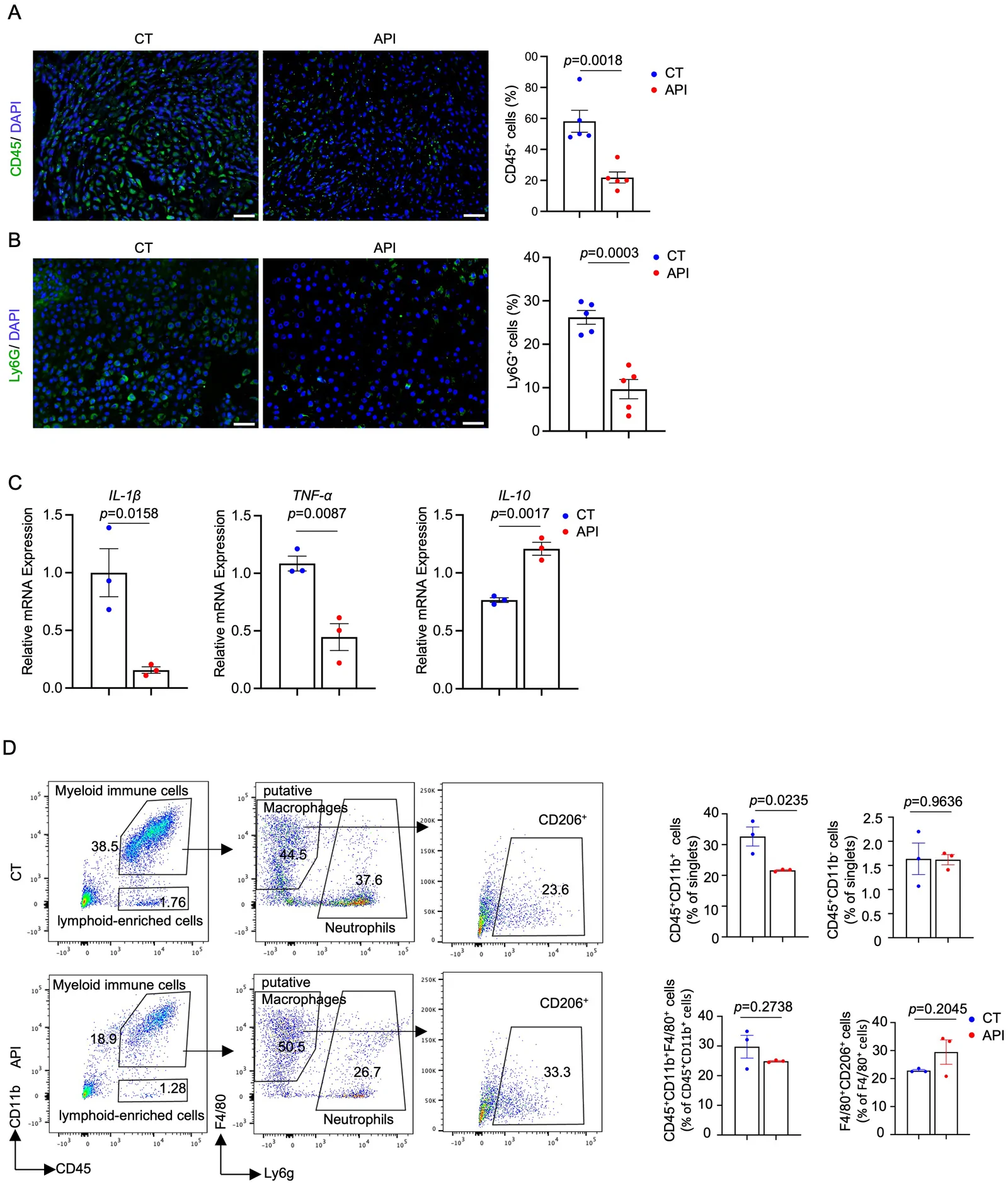
API reduces inflammatory responses in the fracture callus. **(A)** Representative immunofluorescence images and quantitative analysis of CD45^+^ cells in the fracture callus on post-fracture day 7 (green, CD45; blue, DAPI; n = 5). **(B)** Representative immunofluorescence images and quantitative analysis of Ly6G^+^ cells in the fracture callus on day 7 (green, Ly6G; blue, DAPI; n = 5). **(C)** RT-qPCR analysis of *Il1b*, *Tnf*, and *Il10* expression in fracture callus tissue on day 7 (n = 3). **(D)** Representative flow-cytometric gating and quantification of immune-cell populations in day-7 fracture callus from mice in which treatment was initiated on post-fracture day 5. CD45^+^CD11b^+^ and CD45^+^CD11b^-^ cells were quantified as percentages of singlets, F4/80^+^Ly6G^-^ macrophage-enriched cells as a percentage of CD45^+^CD11b^+^ cells, and F4/80^+^CD206^+^ cells as a percentage of F4/80^+^ cells (n = 3). Data are presented as mean ± SEM. Statistical significance was assessed using an unpaired two-tailed Student’s *t*-test. Scale bars, 50 μm.

Flow-cytometric analysis of callus tissues collected on day 7 showed that API treatment initiated on post-fracture day 5 reduced the proportion of CD45^+^CD11b^+^ myeloid cells. In contrast, the proportions of CD45^+^CD11b^-^ lymphoid-enriched cells, F4/80^+^Ly6G^-^ macrophage-enriched cells, and F4/80^+^CD206^+^ M2-like macrophages were not significantly different between groups (**Figure 3D** and **Supplementary Figure 4**). Consistent with these findings, B220^+^ B cells and CD3^+^ T cells were rarely detected by immunofluorescence in either group (**Supplementary Figure 4**). Our findings are consistent with previous studies (41).

We also performed flow-cytometric analysis of callus tissues from mice in which API treatment was initiated on post-fracture day 2 and obtained similar results (**Supplementary Figure 4**). In these samples, *Il1b* and *Tnfa* expression was reduced, whereas *Il10* expression was increased (**Supplementary Figure 4**). Together, these findings (**Figure 3** and **Supplementary Figure 4**) indicate that API treatment initiated on post-fracture day 2 or 5 suppresses myeloid-cell accumulation—likely including neutrophils—and reduces inflammatory gene expression.

### 3.6 API promotes endochondral ossification *in vivo*

To further examine the *in vivo* effects of API on fracture healing, a multi-stage histological and molecular analysis was performed. During the early phase (day 7 post-injury), there was a significant increase in PCNA^+^/SOX9^+^ cells (**Figure 4**), indicating enhanced cell proliferation and chondrocyte differentiation. By day 21, expression of the osteogenic transcription factor RUNX2 was markedly elevated (**Figure 4**, P = 0.0042), along with more than a twofold increase in the mature bone matrix marker osteocalcin (OCN, P = 0.0108) (**Figure 4**). These histological changes were consistent with RT-qPCR data, which showed significant upregulation of chondrogenic markers at day 7 and osteogenic markers at day 14 (**Figure 4**).

**Figure 4:**
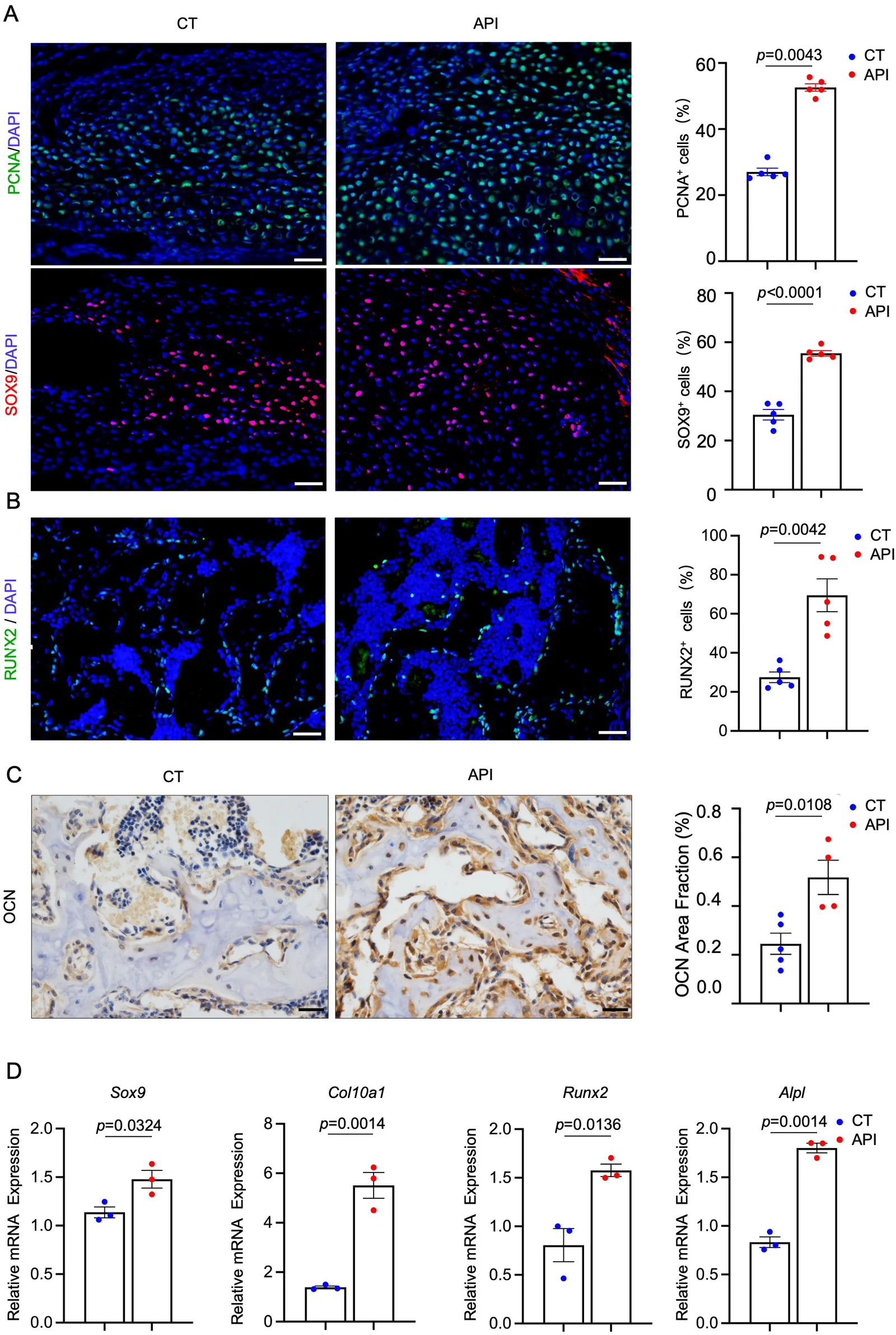
API enhances cell proliferation and chondrogenesis in the fracture callus. **(A)** Representative immunofluorescence staining for the proliferation marker PCNA and chondrogenic marker SOX9 in fracture callus. Quantitative analysis showed significantly higher percentages of PCNA^+^ cells (P = 0.0043) and SOX9^+^ cells (P < 0.0001) in the API group than in the CT group (n = 5). **(B)** Immunofluorescence staining for RUNX2, showing a significantly greater proportion of RUNX2^+^ cells in the API group (P = 0.0042; n =5). **(C)** Immunohistochemical staining for osteocalcin (OCN), showing an increased OCN-positive area fraction after API treatment (P = 0.0108; n = 5). **(D)** RT-qPCR analysis of *Sox9*, *Col10a1*, and *Runx2* expression in fracture callus (n = 3). Data are presented as mean ± SEM. Statistical significance was assessed using an unpaired two-tailed Student’s *t*-test. Scale bars: 50 μm **(A, B)** and 20 μm **(C)**.

### 3.7 API promotes chondrogenic differentiation of BMSCs *in vitro*

Mesenchymal stem cells were then isolated from mouse bone marrow, and a CCK-8 assay was used to evaluate the effects of API on cell viability. At 1 μM, API showed no detectable cytotoxicity and increased the CCK-8 signal relative to vehicle-treated cells (**Figure 5**). To assess the effects of API on BMSC differentiation under non-inflammatory conditions, chondrogenic differentiation assays were performed. Alcian blue staining showed larger and more intensely stained extracellular matrix in the API group, consistent with enhanced chondrogenic differentiation (**Figure 5**). qPCR showed that API significantly increased *Sox9* and *Col10a1* expression. *Runx2* expression was also increased (**Figure 5**). These findings suggest that the effect of API on BMSC differentiation was more consistently reflected in chondrogenic than osteogenic markers. This pattern is consistent with the greater therapeutic effect of API in the femoral fracture model, which involves endochondral ossification, compared with the calvarial defect model, which heals primarily through intramembranous ossification (**Figure 1** and **Supplementary Figure 2**).

**Figure 5:**
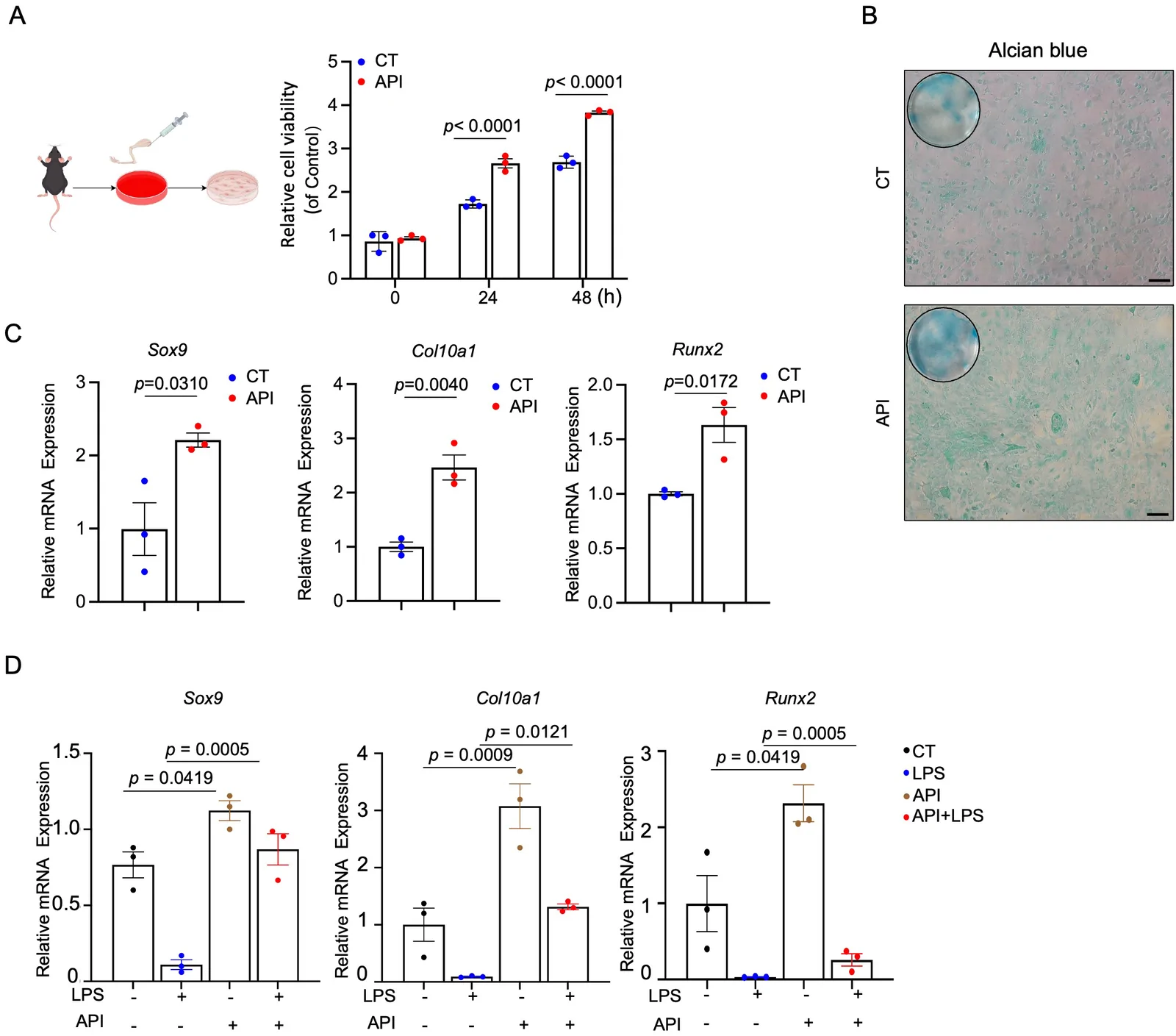
API promotes chondrogenic differentiation and attenuates LPS-associated suppression of chondrogenic markers *in vitro*. **(A)** Schematic of primary cell isolation and experimental timeline. CCK-8 assays were used to assess relative cell viability after API treatment for 24 and 48 h (n =3, P < 0.0001). **(B)** Representative Alcian blue staining of BMSCs cultured under chondrogenic conditions with or without API. Scale bar: 100 μm. **(C)** Under basal conditions, API significantly increased *Sox9*, *Col10a1*, and *Runx2* mRNA expression (n = 3). **(D)** RT-qPCR analysis of *Sox9*, *Col10a1*, and *Runx2* expression under LPS-induced inflammatory conditions. LPS suppressed the expression of all three genes, whereas API co-treatment significantly attenuated these reductions (n = 3). Data are presented as mean ± SEM. Comparisons between two groups were performed using an unpaired two-tailed Student’s *t*-test, and factorial comparisons were performed using two-way ANOVA followed by Tukey’s *post hoc* test.

### 3.8 API attenuates LPS-associated suppression of BMSC differentiation markers

Previous studies have shown that inflammatory cytokines suppress chondrogenic or osteogenic differentiation of BMSCs, likely via activating NF-κB signaling (42–44). Given the inflammatory microenvironment during fracture repair, we next examined the effects of API during BMSC differentiation in the presence of LPS. Under chondrogenic conditions, LPS markedly reduced *Sox9* and *Col10a1* expression, whereas API attenuated these reductions (**Figure 5**). Under osteogenic conditions, LPS also markedly reduced *Runx2* expression, and API significantly attenuated this reduction (**Figure 5**). These findings indicate that API partially counteracted the inhibitory effects of LPS on BMSC differentiation.

### 3.9 The effects of API are mediated by NF-κB signaling inhibition

Consistent with the transcriptomic evidence that API suppresses NF-κB activation, immunofluorescence and Western blot analyses showed a marked decrease in phosphorylated p65 (p-p65) in fracture callus tissue after API treatment, indicating effective NF-κB inhibition (**Figure 6A, B**).

**Figure 6:**
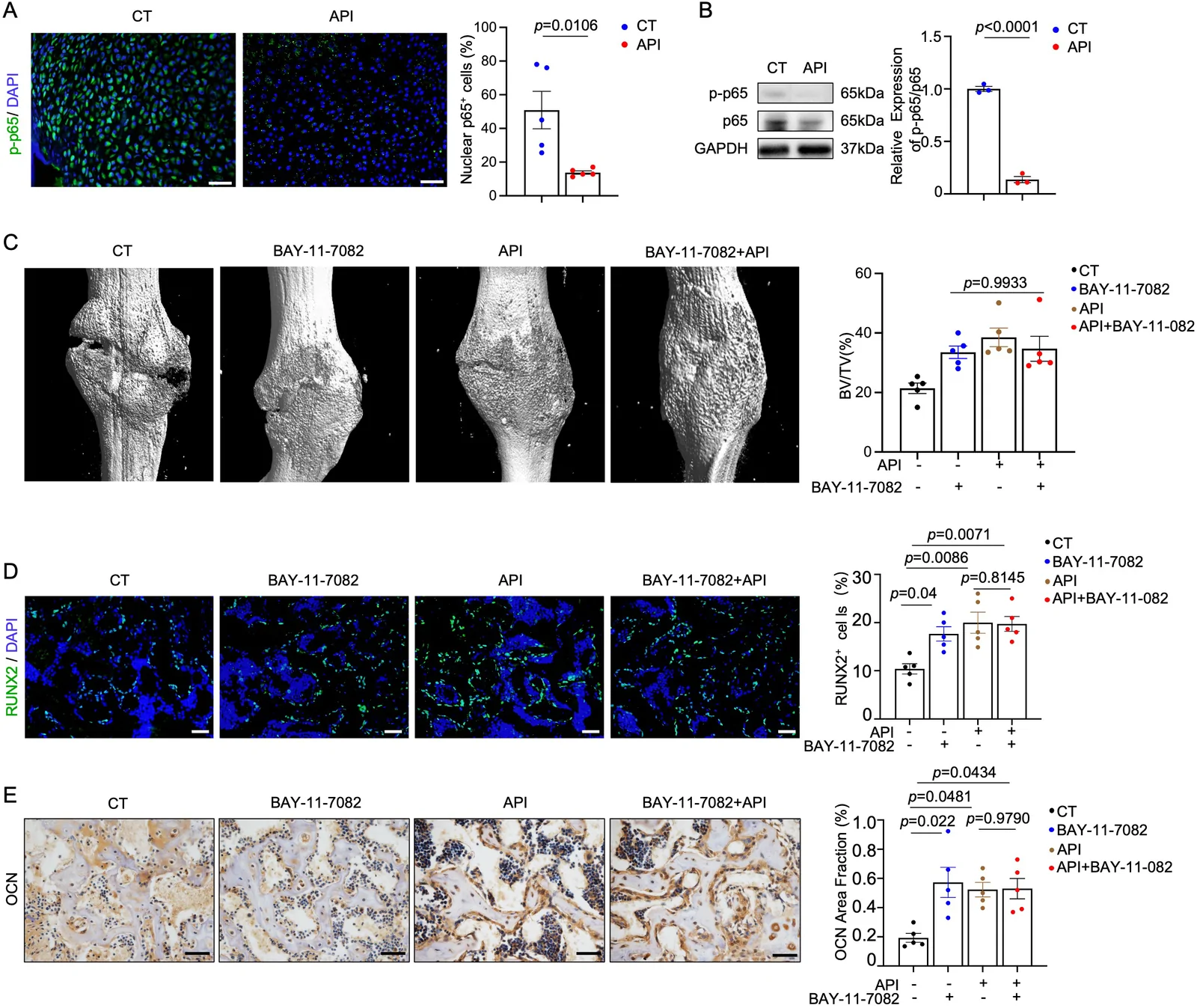
NF-κB inhibition contributes to the effects of API on fracture repair. **(A)** Representative immunofluorescence staining and quantification of nuclear p-p65 in fracture callus. API treatment reduced the percentage of nuclear p-p65^+^ cells compared with CT (P = 0.0106; n = 5). **(B)** Western blotting and quantitative analysis of p-p65 and total p65 showed a significantly decreased p-p65/total p65 ratio in the API group compared with CT (P < 0.0001; n = 3). **(C)** Micro-CT reconstructions of fracture callus from CT, BAY 11-7082, API, and BAY 11-7082 + API groups. BV/TV analysis showed no significant difference between API alone and the combination treatment (P = 0.9933), suggesting no clear additional benefit of the combined treatment (n = 5). **(D)** Immunofluorescence staining for RUNX2 showed increased percentages of RUNX2^+^ cells in the API- and BAY 11-7082-treated groups (n = 5). **(E)** Immunohistochemical staining for OCN showed increased OCN-positive area fractions in the treatment groups compared with CT, with no clear additional increase after combined API and BAY 11–7082 treatment compared with API alone (n = 5). Data are presented as mean ± SEM. Comparisons between two groups were performed using an unpaired two-tailed Student’s *t*-test, and factorial comparisons were performed using two-way ANOVA followed by Tukey’s *post hoc* test. Scale bars: 50 μm **(A, D)** and 20 μm **(E)**.

To examine whether NF-κB inhibition contributes to the fracture-healing effects of API, BAY 11–7082 was evaluated in the femoral fracture model. *In vivo* micro-CT analysis at day 21 post-fracture revealed that BAY 11-7082 alone or in combination with API similarly increased callus BV/TV compared with controls, a finding supported by histomorphometric evaluation (**Figure 6C**). Immunofluorescence for RUNX2 and immunohistochemistry for OCN showed increased expression of these osteogenic-associated markers in the treated groups (**Figure 6D, E**). The similar responses observed with BAY 11–7082 alone and in combination with API are consistent with convergence of the two treatments on NF-κB-related mechanisms, although these data do not exclude the involvement of other pathways.

### 3.10 *In silico* and cellular studies support an interaction between API and IKKβ

An earlier study used the crystal structures of calcium-dependent protein kinase (CDPK) from *Toxoplasma gondii* (PDB ID: 3IS5) and protein kinase A (PKA) from *Bos taurus* (PDB ID: 1Q61) as templates to model molecular docking between apigenin and IKKα/IKKβ, and suggested that apigenin interacts with IKKα to inhibit NF-κB signaling (45), However, because these models were based on unrelated kinase structures, they may not accurately represent the API–IKK interaction. We focused on IKKβ because it plays a more critical role in canonical NF-κB signaling than IKKα (46) (**Figure 7**). Molecular docking predicted a favorable binding pose of API within IKKβ, with a docking score of −9.3 kcal/mol. API was predicted to form hydrogen bonds with Cys91 and Asp95, together with additional hydrophobic and van der Waals interactions (**Figure 7**).

**Figure 7:**
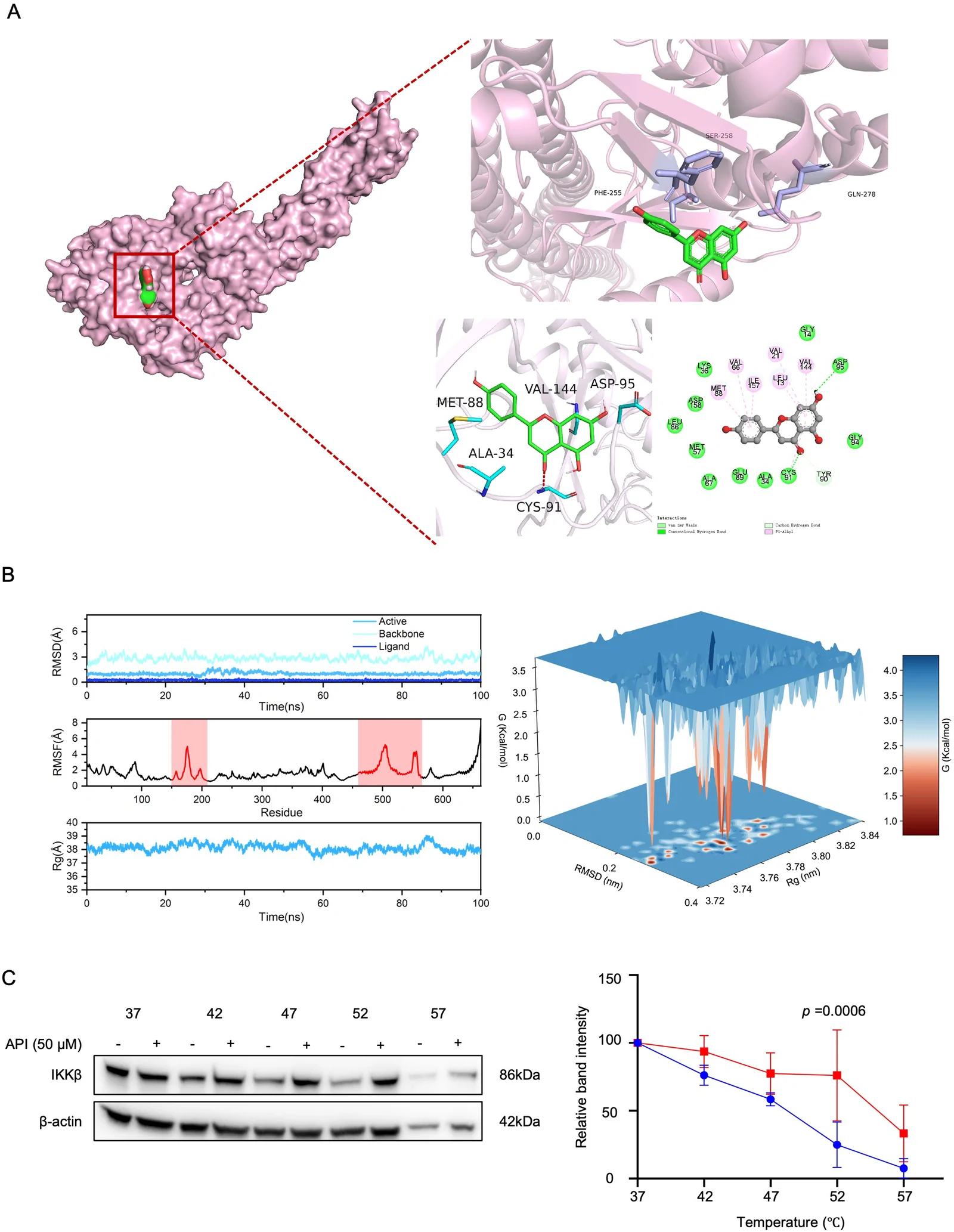
*In silico* and cellular analyses support an interaction between API and IKKβ. **(A)** Predicted binding pose of API within the IKKβ structure (PDB ID: 4KIK), showing hydrogen bonds with Cys91 and Asp95 and hydrophobic contacts with Met88 and Val144. **(B)** Molecular dynamics analysis of the predicted API–IKKβ complex over 100 ns. RMSD gradually approached a relatively stable level toward the end of the simulation, while RMSF and radius of gyration (Rg) analyses were used to assess residue flexibility and protein compactness. **(C)** Cellular thermal shift assay of IKKβ in RAW264.7 cell lysates incubated with vehicle or API (50 μM). Soluble IKKβ abundance was normalized to the corresponding unheated or 37 °C sample. Greater retention of soluble IKKβ was detected after API treatment at 52 °C, whereas no significant difference was observed at the other temperatures tested (*n* = 3). Data are presented as mean ± SD and were analyzed using two-way ANOVA followed by Tukey’s *post hoc* test.

We also performed molecular dynamics simulations to assess the stability of the API–IKKβ complex. The RMSD gradually stabilized and reached a relatively stable level toward the end of the 100-ns simulation. The radius of gyration changed only slightly, while RMSF analysis showed relatively low fluctuations across most residues, with greater flexibility mainly around residues 160 and 500. The main hydrogen bond showed an occupancy of 98.68%, with an average bond length of 2.94 Å and an average angle of 156.6° (**Figure 7**). These results further supported the stability of the predicted API–IKKβ complex.

We also performed a cellular thermal shift assay (CETSA) to assess the interaction between API and IKKβ in RAW264.7 cells, a monocyte/macrophage cell line. Soluble IKKβ levels gradually decreased with increasing temperature in both vehicle- and API-treated lysates. However, at 52 °C, significantly more IKKβ remained in the soluble fraction of API-treated lysates than in vehicle-treated lysates (P = 0.0006; **Figure 7**). These findings provide additional evidence that API may interact with IKKβ, although direct binding between API and IKKβ requires further validation.

### 3.11 IKKβ is involved in API-induced chondrogenic differentiation of BMSCs

Finally, we examined the role of IKKβ in the API-induced chondrogenic response of BMSCs. We first assessed the effects of API on osteogenic and chondrogenic differentiation. Under osteogenic induction conditions, API had little effect on the expression of the osteogenic markers *Osx* and *Alpl*, although *Runx2* expression was increased (**Supplementary Figure 5A**). Notably, RUNX2 is required for both chondrogenic and osteogenic differentiation. These findings suggest that API does not substantially promote osteogenic differentiation of BMSCs, consistent with its lack of therapeutic effect in the calvarial repair model. In contrast, under chondrogenic induction conditions, API increased Alcian blue staining and the expression of *Sox9* and *Col10a1* in control siRNA-transfected cells (**Supplementary Figure 5C**). BMSCs were then transfected with siRNA targeting *Ikbkb*, and RT–qPCR and Western blotting confirmed marked reductions in *Ikbkb* mRNA and IKKβ protein levels relative to cells transfected with control siRNA (**Supplementary Figure 5E**). *Ikbkb* knockdown similarly increased Alcian blue staining and the expression of *Sox9* and *Col10a1*. However, API treatment did not further enhance Alcian blue staining or chondrogenic-marker expression in *Ikbkb*-knockdown cells (**Supplementary Figure 5C**). These findings suggest that IKKβ contributes to the chondrogenic effects of API in BMSCs.

## 4 Discussion

Delayed union and non-union fractures remain major clinical challenges (47, 48). In this study, apigenin (API), a flavonoid found in Epimedium, celery, and other plants, improved fracture healing in a timing-dependent manner, with the clearest therapeutic effect observed when treatment was initiated during the transition from inflammation resolution to cartilage formation. This response was accompanied by reduced IKKβ/NF-κB signaling and inflammatory activity at the fracture site. In addition, API promoted chondrogenic differentiation of BMSCs *in vitro*, suggesting that its effects may involve both modulation of the inflammatory microenvironment and regulation of stromal cell differentiation (**Figure 8**).

**Figure 8:**
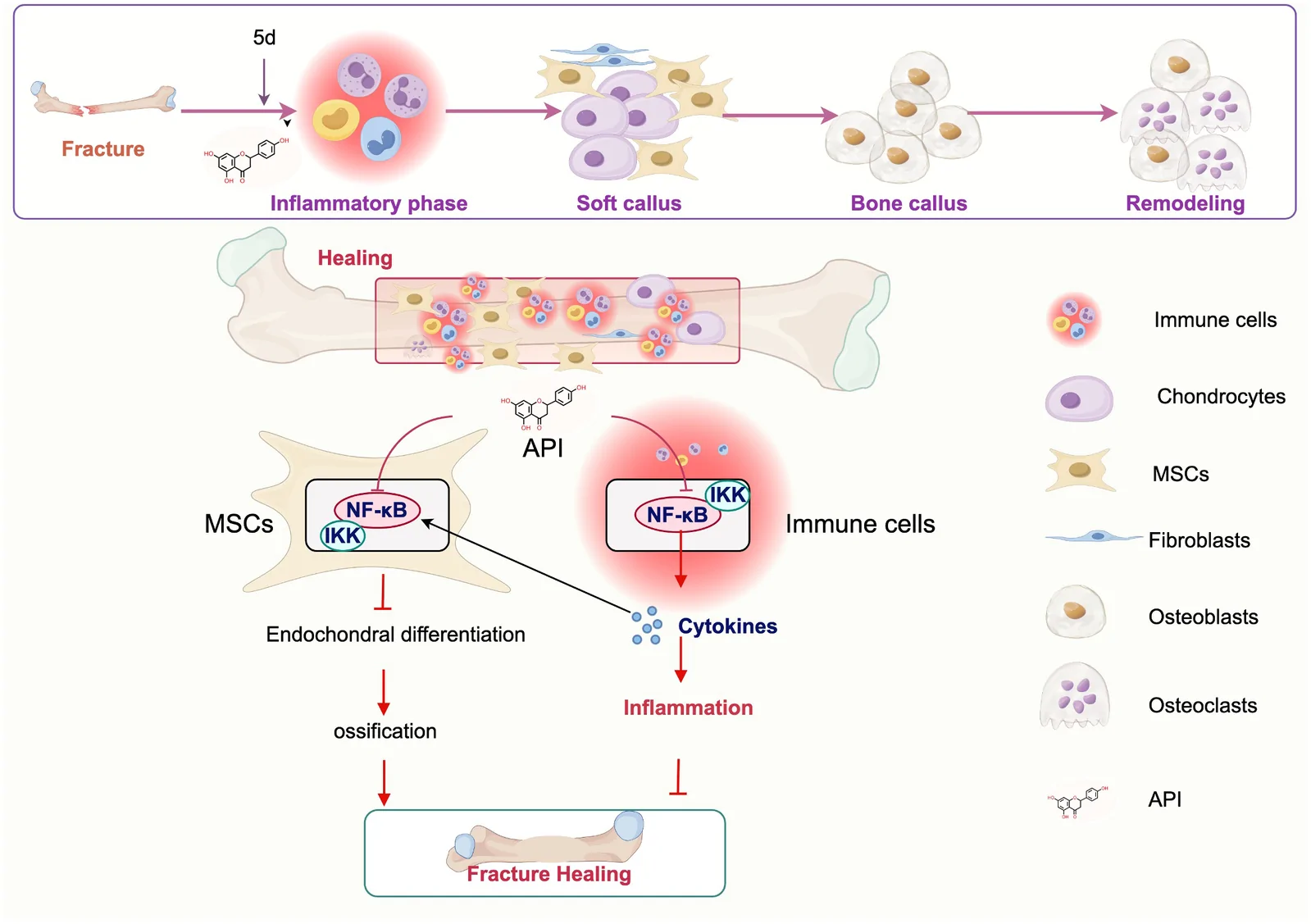
Schematic diagram of the molecular mechanisms by which API promotes fracture healing. .

Given the limited systemic bioavailability of many natural flavonoids, API was administered locally to increase exposure at the fracture site. The effects of API varied markedly according to treatment timing. Administration beginning on day 1 or 2 did not improve fracture healing, whereas treatment initiated on day 5 produced clear radiographic and micro-CT improvements. Because the early inflammatory response is required for debris clearance, recruitment of reparative cells, and initiation of callus formation (49), excessive suppression of inflammation during this phase may be unfavorable.

Flow-cytometric analysis indicated that myeloid cells, likely neutrophils, are the predominant immune cells during early fracture healing. These findings are broadly consistent with previous studies (41). API treatment initiated on post-fracture day 2 or 5 significantly reduced myeloid-cell abundance and expression of the inflammatory cytokines *Il1b* and *Tnfa*. However, only API treatment initiated on day 5, but not on day 1 or 2, improved fracture healing. This time point coincides with the resolution of inflammation and onset of cartilage formation. Therefore, the therapeutic effects of API may depend on its timing relative to the transition from inflammation to chondrogenesis.

These findings suggest a potential link between the immune and stromal compartments. By reducing myeloid-cell accumulation and inflammatory signaling within the callus, API may create a local environment that is more permissive for chondrogenic commitment. In parallel, inhibition of IKKβ/NF-κB signaling in BMSCs may directly reinforce this differentiation response.

Several lines of evidence support the involvement of NF-κB signaling in the effects of API. Transcriptomic analysis showed suppression of NF-κB signaling, whereas JAK–STAT signaling was not comparably altered. This finding was accompanied by reduced p-p65 levels in fracture callus tissue. BAY 11–7082 reproduced several effects of API, and combined treatment with BAY 11–7082 and API did not provide a clear additional benefit. Together, these findings suggest that inhibition of NF-κB signaling substantially contributes to the API response, although the involvement of additional pathways cannot be excluded.

API also influenced BMSC differentiation. Under chondrogenic induction conditions, API increased Alcian blue staining and expression of *Sox9* and *Col10a1*. Silencing *Ikbkb* produced a similar response, whereas subsequent API treatment did not further enhance chondrogenic differentiation. These findings suggest that IKKβ contributes to the chondrogenic response of BMSCs to API. In contrast, API did not significantly promote osteogenic differentiation in either the calvarial repair model or *in vitro* BMSC osteogenic-differentiation assays. Overall, the effects of API on BMSC differentiation were more consistently reflected by chondrogenic than osteogenic markers.

The differing responses observed in the femoral-fracture and calvarial-defect models are consistent with a greater effect of API on endochondral bone repair. API improved callus formation and fracture bridging in the femoral fracture model, in which cartilage formation is an essential intermediate step. In contrast, API had little effect on calvarial defect repair or on RUNX2 and OCN expression in this model, which heals primarily through intramembranous ossification (50). These findings suggest that API is not a general stimulator of bone formation. Instead, its beneficial effects in the femoral-fracture model may be related to the formation and subsequent ossification of the cartilaginous callus. Direct comparison of the molecular responses in the two models will be needed to investigate this possibility further.

Our structural and cellular findings provide additional evidence supporting the involvement of IKKβ. Molecular docking using the IKKβ crystal structure predicted a favorable binding pose for API, with a docking score of −9.3 kcal/mol and hydrogen-bond interactions with Cys91 and Asp95. Molecular dynamics simulations further indicated that the predicted API–IKKβ complex remained relatively stable over 100 ns. This approach differs from earlier studies in which IKKα and IKKβ were modeled using unrelated kinase structures as templates (45). Our CETSA results showed greater retention of soluble IKKβ following API treatment, supporting a potential interaction between API and IKKβ. However, direct biochemical binding assays are needed to characterize this interaction more precisely. Together, the transcriptomic, inhibitor, knockdown, docking, and CETSA data support a role for IKKβ/NF-κB signaling in mediating the effects of API on inflammation and chondrogenic differentiation. Nevertheless, additional molecular targets may also contribute to the effects of API.

Several limitations should be acknowledged. First, the *in vivo* studies were conducted in young, healthy mice; therefore, the effects of API should be evaluated in aged, osteoporotic, and impaired-healing models. Second, the large gap between the tested doses and the absence of intermediate doses limited characterization of the dose–response relationship and identification of the minimum effective dose. Finally, although our findings support the involvement of IKKβ/NF-κB signaling, they do not establish IKKβ as the sole target of API. Direct binding assays and investigation of related pathways, including Wnt/β-catenin and MAPK signaling, are warranted.

## 5 Conclusion

Our findings indicate that the therapeutic effects of apigenin on fracture repair are highly dependent on treatment timing. When administered during the transition from inflammation to cartilage formation, apigenin suppressed IKKβ/NF-κB signaling, reduced inflammatory responses, and enhanced chondrogenic differentiation (**Figure 8**). Pharmacological inhibition and *Ikbkb* knockdown further support a functional role for the IKKβ/NF-κB axis in this response, whereas docking, molecular dynamics, and thermal-shift analyses provide supportive evidence for an interaction between apigenin and IKKβ. Together, these effects appear to favor endochondral fracture repair rather than broadly stimulate osteogenic differentiation.

## Data Availability

The datasets presented in this study can be found in online repositories. The names of the repository/repositories and accession number(s) can be found below: GSE325505 (GEO).”- not publicly available.

## Supplementary material

Supplementary Figure 1 Effects of API administration timing and dosage on fracture healing. **(A)**. Representative X-ray images and histological staining of mice treated with 45 ng/kg API initiated at day 5 post-fracture (*n* = 5). **(B)**. Representative X-ray images and histological staining of fracture calluses from mice treated with 5 mg/kg API initiated immediately after surgery (Day 1) (*n* = 5). **(C)**. Expression of the chondrogenic genes *Sox9* and *Col10a1* and the osteogenic genes *Runx2* and *Alpl* in the same samples (*n* = 3). **(D)**. Apigenin concentrations in fracture callus were determined by LC–MS/MS at 2, 4, 8, and 24 h following local administration of API at 5 mg/kg. Samples at each time point were collected from independent animals (n = 3 per time point).

Supplementary Figure 2 API exerts minimal osteogenic effects on membranous ossification in a calvarial defect model. **(A)**. Schematic diagram illustrating the experimental design. Calvarial defects were created in mice, with API administration initiated on postoperative day 5. **(B)**. Micro-CT assessment of bone regeneration. Representative 3D surface renderings (left) show persistent defect patency at 4, 6, and 8 weeks. Quantitative analysis of defect diameter (right) showed only minor closure in the API-treated group compared with controls (n = 3–4 mice per group). **(C)**. Representative immunofluorescence staining and quantitative analysis of RUNX2 and OCN in the defect region. No significant differences in RUNX2 or OCN staining were detected between the CT and API groups (n = 3 mice per group). Data are presented as mean ± SEM. Scale bars: 50 μm.

Supplementary Figure 3 Characteristics of the transcriptional profile of early fracture callus. **(A)**. KEGG and GO enrichment analyses of upregulated and downregulated genes in API-treated callus versus control. **(B)**. Heatmap showing the expression patterns of selected JAK–STAT pathway-related genes in day-7 fracture callus (n = 3 biological replicates per group).

Supplementary Figure 4 Immune-cell characterization of day-7 fracture callus following API treatment initiated at different post-fracture time points. **(A)**. Representative flow-cytometric gating strategy. P1 was gated based on FSC-A and SSC-A to exclude debris, followed by FSC-A/FSC-H gating to identify singlets (P2) and exclude doublets. **(B)**. Representative immunofluorescence staining of B220^+^ and CD3^+^ cells in day-7 fracture callus. **(C)**. Representative flow-cytometric plots and quantification of CD45^+^CD11b^+^ myeloid cells, CD45^+^CD11b^-^ lymphoid-enriched cells, F4/80^+^Ly6G^-^ macrophage-enriched cells, and F4/80^+^CD206^+^ macrophages in day-7 fracture callus from mice in which API treatment was initiated on post-fracture day 2 (n = 3 mice per group). **(D)**. RT-qPCR analysis of *Il1b*, *Tnf*, and *Il10* expression in day-7 fracture callus. Data are presented as mean ± SEM and were analyzed using an unpaired two-tailed Student’s *t*-test. Scale bars: 50 μm.

Supplementary Figure 5 Effects of API and *Ikbkb* knockdown on chondrogenic and osteogenic differentiation of BMSCs. **(A)**. Representative ALP staining of BMSCs in the CT and API groups. **(B)**. RT-qPCR analysis of Osx and Alpl expression in control and API-treated groups. **(C)**. RT-qPCR analysis of *Ikbkb* mRNA expression after siRNA-mediated knockdown, confirming efficient silencing. **(D)**. Western blot analysis of IKKβ protein levels after siRNA-mediated knockdown, confirming efficient silencing. **(E)**. Alcian blue staining of BMSCs cultured under chondrogenic conditions for 14 days. API treatment (1 μM) increased Alcian blue staining compared with vehicle control, whereas *Ikbkb* knockdown produced a similar response. Addition of API after *Ikbkb* knockdown did not produce a further clear increase. **(F)**. RT-qPCR analysis of the chondrogenic marker genes *Sox9* and *Col10a1* in *BMSCs* under chondrogenic conditions. API treatment and *Ikbkb* knockdown increased the expression of both genes, whereas addition of API after *Ikbkb* knockdown produced no further clear increase. Data are presented as mean ± SD (n = 3). Two-group comparisons were performed using an unpaired two-tailed Student’s *t*-test, and four-group comparisons were performed using two-way ANOVA followed by Tukey’s *post hoc* test.

Supplementary Table 1 Primer sequences used for RT-qPCR in this study.
