# Supplementary material for: Apigenin accelerates fracture healing by enhancing endochondral differentiation and inflammation resolution via targeting NF-κB signaling
Source: Front Immunol. 2026 Sep 14;17:1877955. doi: 10.3389/fimmu.2026.1877955 (PMC13616616; doi:10.3389/fimmu.2026.1877955)
Supplement: Supplementary file 1 [file SupplementaryFile1.pdf]

## *Supplementary Material*

**Supplementary Table 1**

| Primer | sequences（5'-3'） |
|---|---|
| *Runx2* | sense 5'-CCGGTCTCCTTCCAGGAT-3' |
| | antisense 5'-GGGAACTGCTGTGGCTTC-3 |
| *Sp7* | sense 5'-GGAAAGGAGGCACAAAGAAGC-3' |
| | antisense 5'- CCCCTTAGGCACTAGGAGC-3 |
| *ALP* | sense 5'- ATGCCCTGAAACTCCAAA -3' |
| | antisense 5'- AGACGCCCATACCATCTC -3' |
| *Col10* | sense 5'- TAAGGGTCCCCAATGGTGAGA -3' |
| | antisense 5'- GGGTCCCTCGACTCCTACAT -3' |
| *Sox9* | sense 5'-AGTCCCAGCGAACGCACATCA-3' |
| | antisense 5'- GTCGTATTGCGAGCGGGTGAT-3' |
| *IL-1β* | sense 5'- TGCCACCTTTTGACAGTGATG-3' |
| | antisense 5'- TGATGTGCTGCTGCGAGATT-3' |
| *IL-10* | sense 5'- GCTCTTACTGACTGGCATGAG-3' |
| | antisense 5'- CGCAGCTOTAGGAGCATGTG-3' |
| *TNF-α* | sense 5'- TAGCCCACGTCGTAGCAAAC-3' |
| | antisense 5'- TGTCTTTGAGATCCATGCCGT-3' |
| *Ikbkb* | sense 5'- ATCAGGCGACAGGTGAACAG-3' |
| | antisense 5'-GGCCACAGCAGTTCTCGAA-3' |
| *GAPDH* | sense 5'-TGACCTCAACTACATGGTCTACA-3' |
| | antisense 5'-CTTCCCATTCTCGGCCTTG-3' |
| *B2M* | sense 5'- GAGGCTATCCAGCGTACTCCA-3' |
| | antisense 5'- CGGCAGGCATACTCATCTTTT-3' |

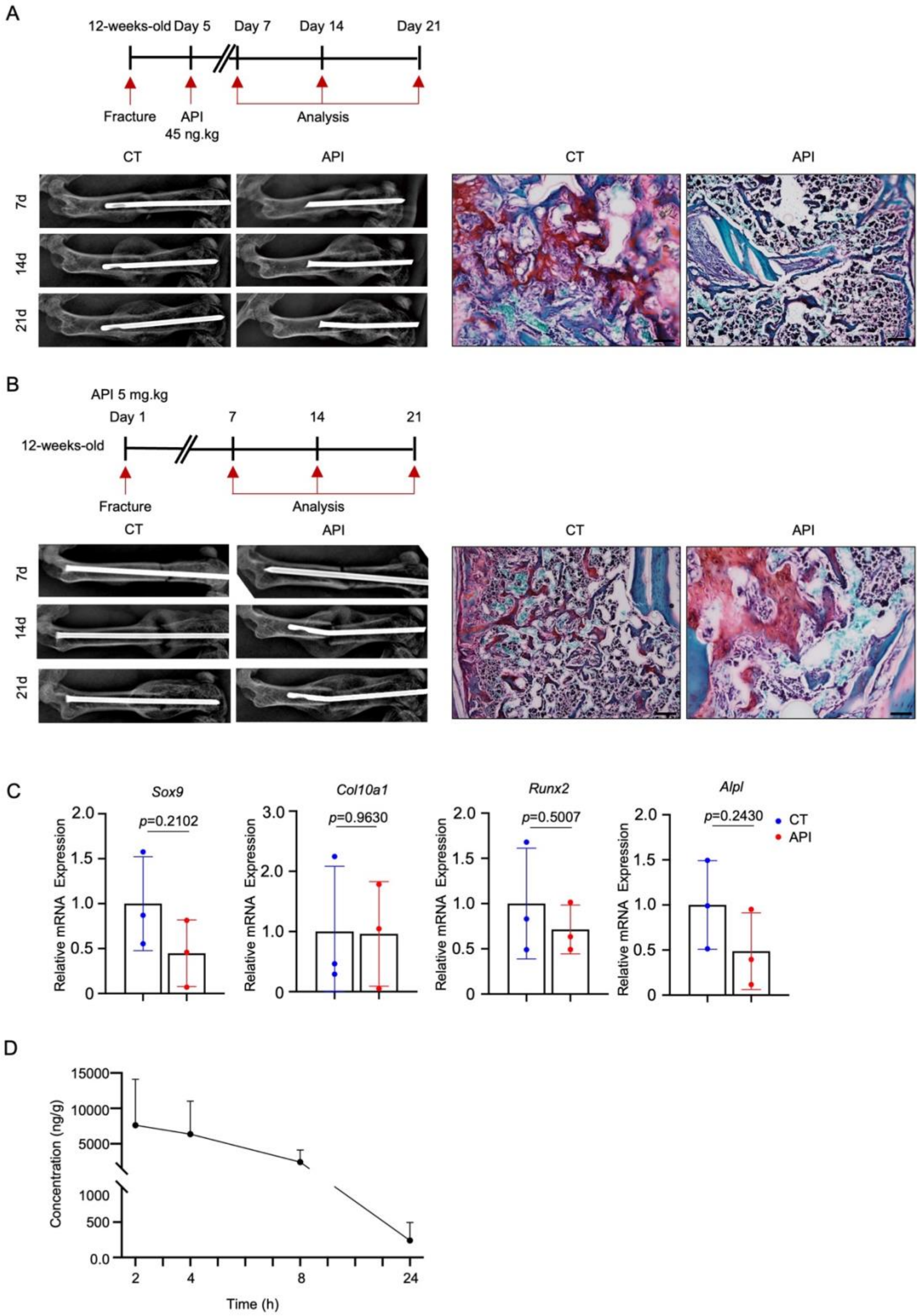


**Supplementary Figure S1. Effects of API administration timing and dosage on fracture healing.** A. Representative X-ray images and histological staining of mice treated with 45 ng/kg API initiated at day 5 post-fracture (*n* = 5). B. Representative X-ray images and histological staining of fracture calluses from mice treated with 5 mg/kg API initiated immediately after surgery (Day 1) (*n* = 5). C. Expression of the chondrogenic genes *Sox9* and *Col10a1* and the osteogenic

genes *Runx2* and *Alpl* in the same samples (*n* =3). D. Apigenin concentrations in fracture callus were determined by LC–MS/MS at 2, 4, 8, and 24 h following local administration of API at 5 mg/kg. Samples at each time point were collected from independent animals (n = 3 per time point).

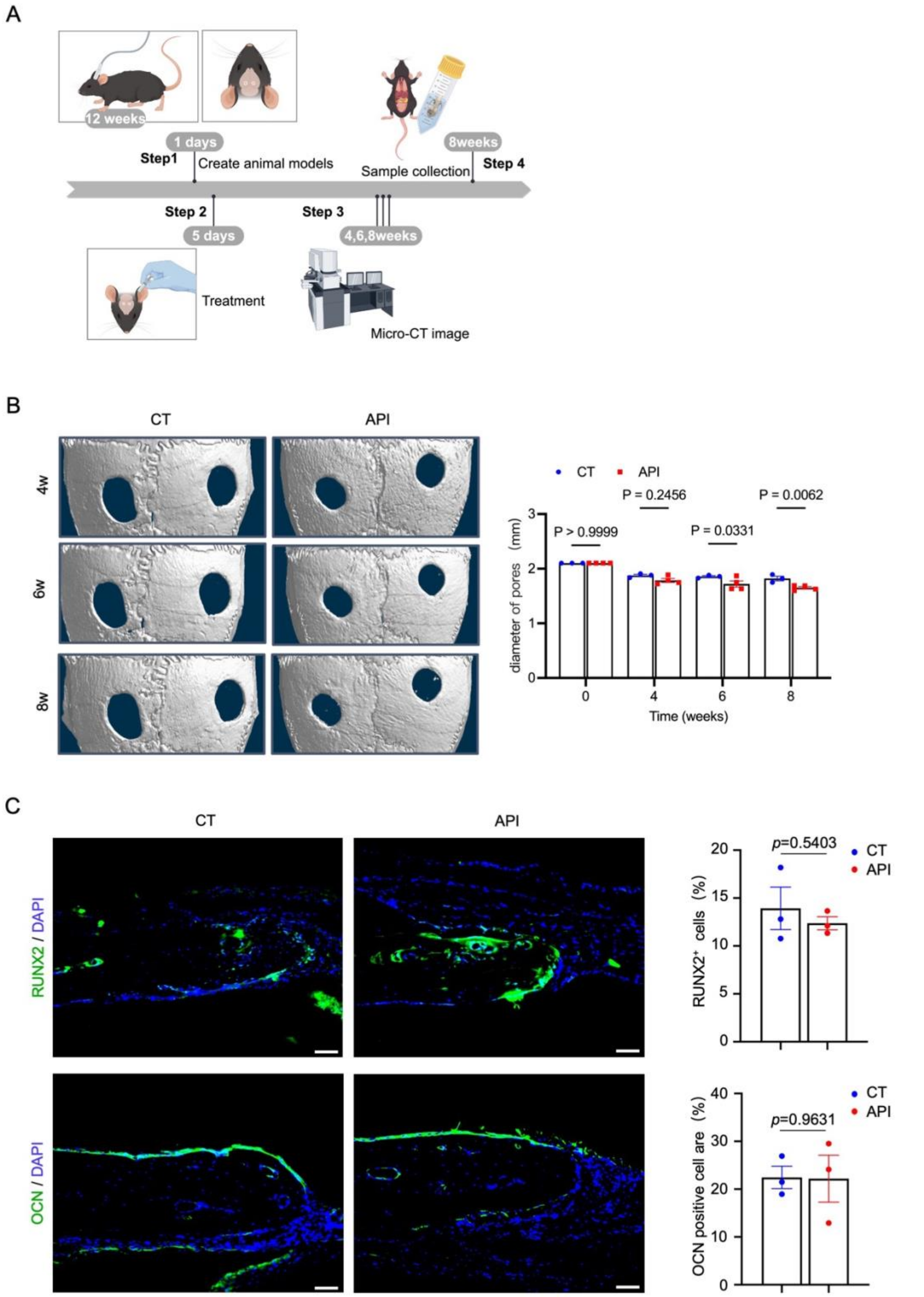


**Supplementary Figure S2. API exerts minimal osteogenic effects on membranous ossification in a calvarial defect model.**

A. Schematic diagram illustrating the experimental design. Calvarial defects were created in mice, with API administration initiated on postoperative day 5. B. Micro-CT assessment of bone regeneration. Representative 3D surface renderings (left) show persistent defect patency at 4, 6, and

8 weeks. Quantitative analysis of defect diameter (right) showed only minor closure in the API-treated group compared with controls (n = 3-4 mice per group). C. Representative immunofluorescence staining and quantitative analysis of RUNX2 and OCN in the defect region. No significant differences in RUNX2 or OCN staining were detected between the CT and API groups (n = 3 mice per group). Data are presented as mean ± SEM. Scale bars: 50 μm.

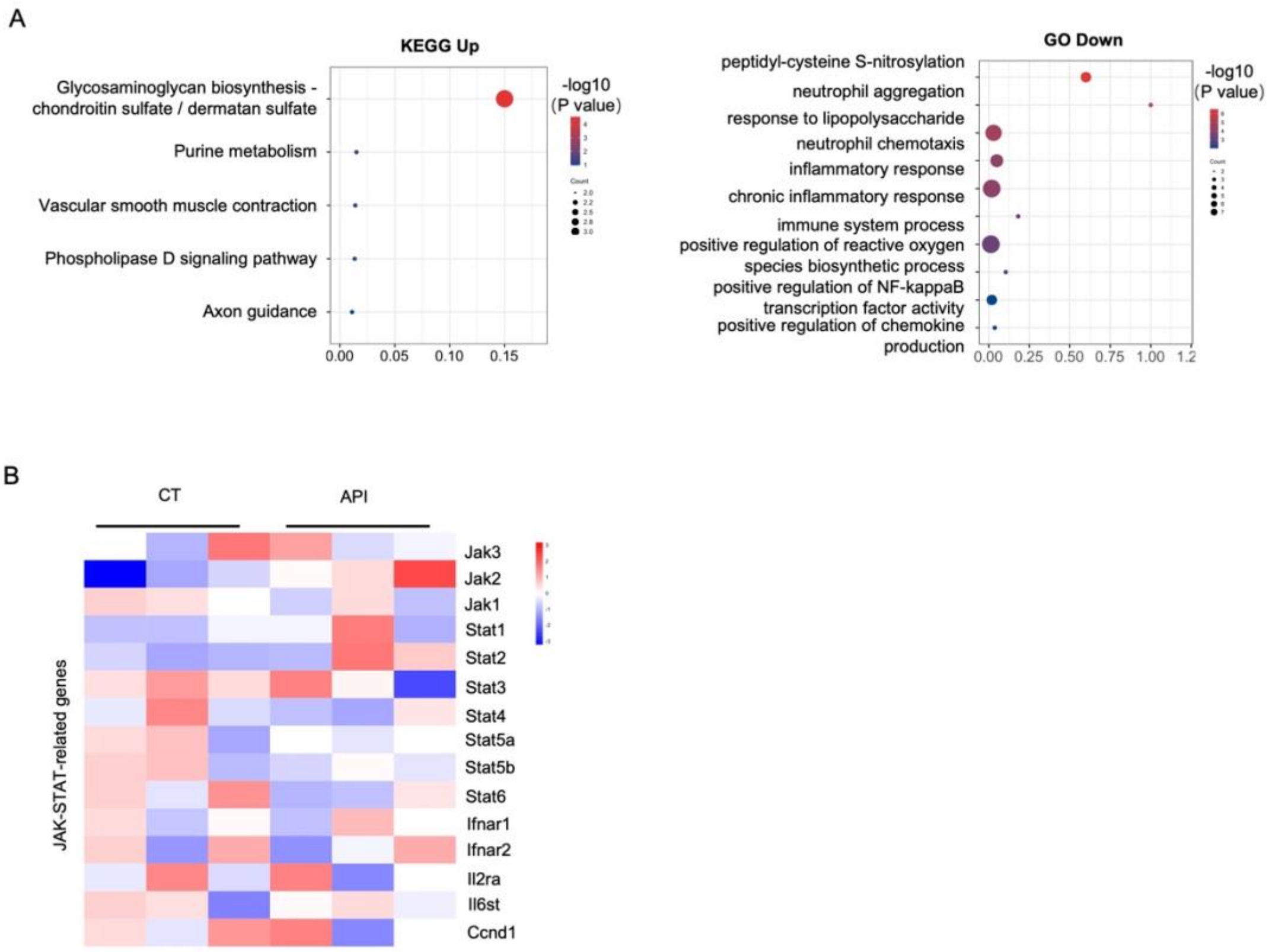


**Supplementary Figure S3. Characteristics of the transcriptional profile of early fracture callus.** A. KEGG and GO enrichment analyses of upregulated and downregulated genes in API-treated callus versus control. B. Heatmap showing the expression patterns of selected JAK–STAT pathway-related genes in day-7 fracture callus (n = 3 biological replicates per group).

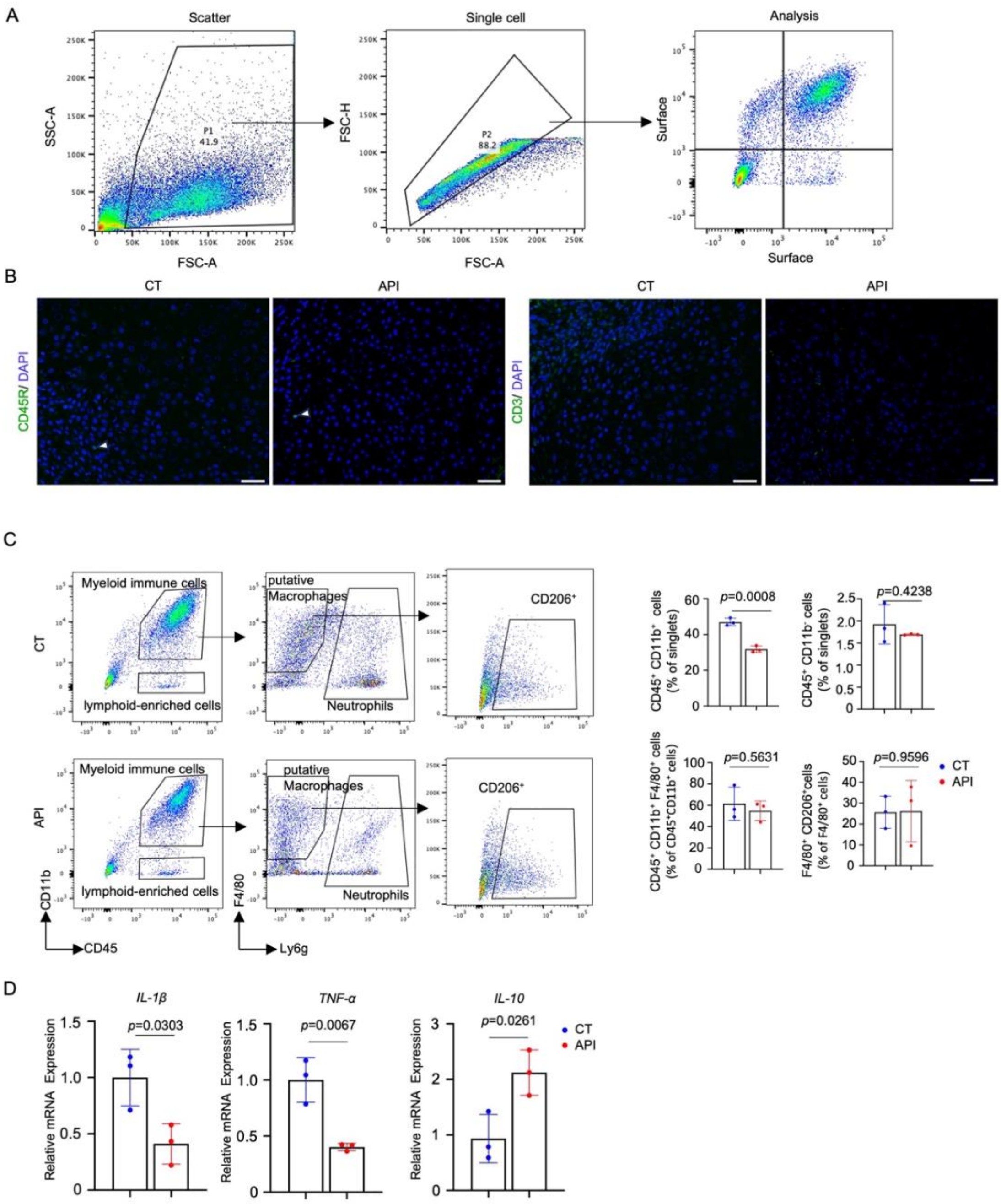


**Supplementary Figure S4. Immune-cell characterization of day-7 fracture callus following API treatment initiated at different post-fracture time points.**

A. Representative flow-cytometric gating strategy. P1 was gated based on FSC-A and SSC-A to exclude debris, followed by FSC-A/FSC-H gating to identify singlets (P2) and exclude doublets. B. Representative immunofluorescence staining of $B220^+$ and $CD3^+$ cells in day-7 fracture callus. C. Representative flow-cytometric plots and quantification of $CD45^+CD11b^+$ myeloid cells, $CD45^+CD11b^-$ lymphoid-enriched cells, $F4/80^+Ly6G^-$ macrophage-enriched cells, and $F4/80^+CD206^+$ macrophages in day-7 fracture callus from mice in which API treatment was initiated on post-fracture day 2 (n = 3 mice per group). D. RT-qPCR analysis of *Il1b*, *Tnf*, and *Il10* expression in day-7 fracture callus. Data are presented as mean ± SEM and were analyzed using an unpaired two-tailed Student's *t*-test. Scale bars: 50 μm.

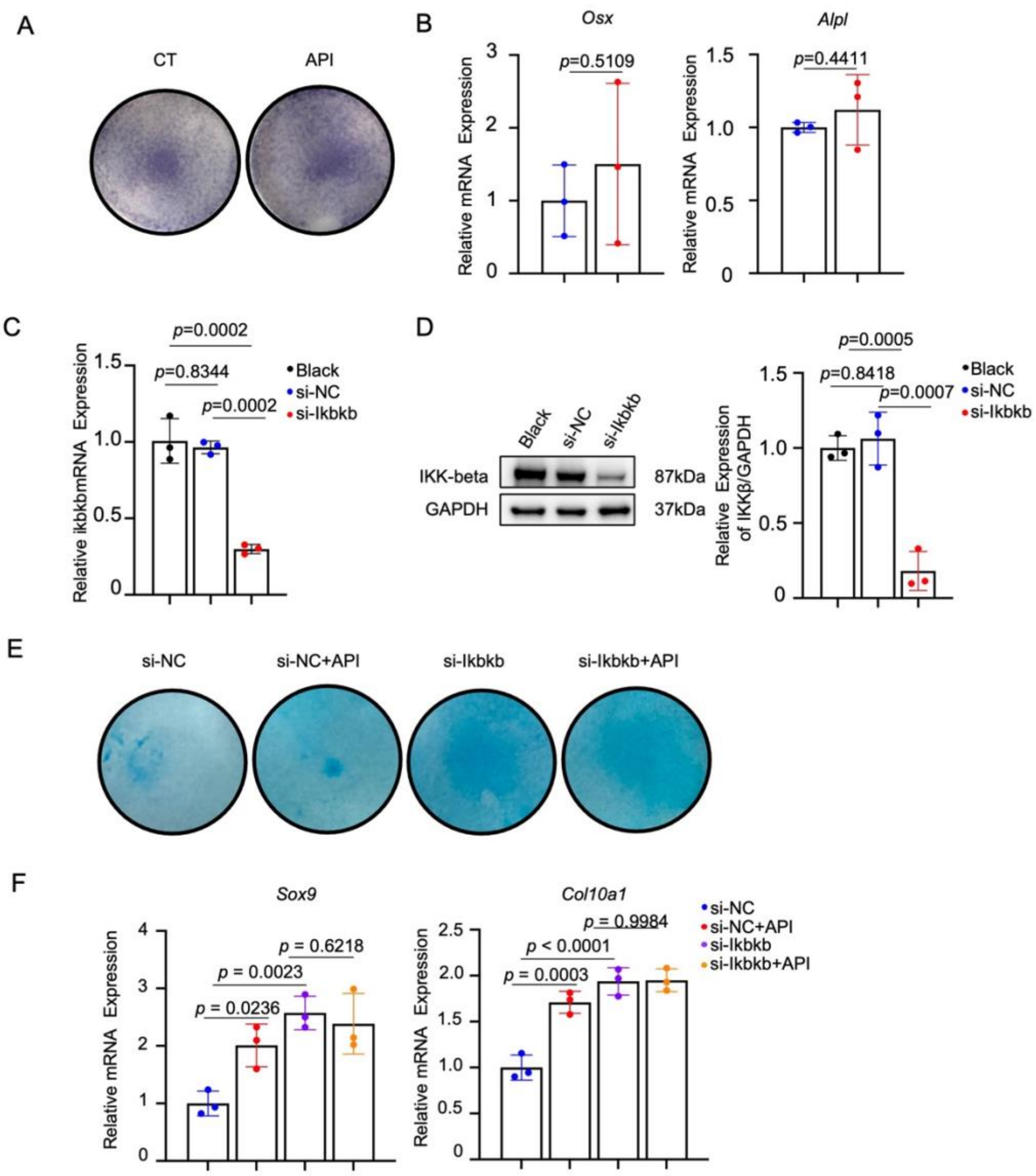


**Supplementary Figure S5. Effects of API and *Ikbkb* knockdown on chondrogenic and osteogenic differentiation of BMSCs.**

A. Representative ALP staining of BMSCs in the CT and API groups. B. RT-qPCR analysis of Osx and Alpl expression in control and API-treated groups. C. RT-qPCR analysis of *Ikbkb* mRNA expression after siRNA-mediated knockdown, confirming efficient silencing. D. Western blot analysis of IKKβ protein levels after siRNA-mediated knockdown, confirming efficient silencing. E. Alcian blue staining of BMSCs cultured under chondrogenic conditions for 14 days. API treatment (1 μM) increased Alcian blue staining compared with vehicle control, whereas *Ikbkb* knockdown produced a similar response. Addition of API after *Ikbkb* knockdown did not produce a further clear increase. F. RT-qPCR analysis of the chondrogenic marker genes *Sox9* and *Col10a1* in *BMSCs* under chondrogenic conditions. API treatment and *Ikbkb* knockdown increased the expression of both genes, whereas addition of API after *Ikbkb* knockdown produced no further clear increase. Data are presented as mean ± SD (n = 3). Two-group comparisons were performed using an unpaired two-tailed Student's *t*-test, and four-group comparisons were performed using two-way ANOVA followed by Tukey's post hoc test.
